# Supplementary material for: Modifying electron transfer between photoredox and organocatalytic units via framework interpenetration for β-carbonyl functionalization
Source: Nat Commun. 2017 Aug 25;8:361. doi: 10.1038/s41467-017-00416-8 (PMC5572462; doi:10.1038/s41467-017-00416-8)
Supplement: Supplementary file 1 — Supplementary Information [file 41467_2017_416_MOESM1_ESM.pdf]

Description of Supplementary Files

File Name: Supplementary Information
Description: Supplementary Figures, Supplementary Tables, Supplementary Methods and Supplementary References

File Name: Supplementary Data 1
Description: Crystal data of complex InP-1.

File Name: Supplementary Data 2
Description: Crystal data of complex InP-2.

File Name: Supplementary Data 3
Description: Crystal data of complex InP-3.

File Name: Supplementary Data 4
Description: Crystal data of complex InP-4.

File Name: Supplementary Data 5
Description: Crystal data of complex InP-5.

File Name: Peer Review File

Supplementary Methods

Materials: Unless otherwise specified, all chemicals were of reagent-grade quality, were obtained from commercial sources and were used without further purification. 4,4',4''-Nitrilotrisbenzoicacid (**H_3NTB**)[1], *L*-/*D*-pyrrolidin-2-ylimidazole (*L*-/*D*-**PYI**)[2], MOF-**150**[3] and Zn-**PYI**1[4] were synthesized according to published procedures.

Measurements: The elemental analyses of C, H and N were performed on a Vario EL III elemental analyzer. FT-IR spectra were recorded from KBr pellets on a JASCO FT/IR-430. The powder XRD (PXRD) diffractograms were obtained on a Rigaku D/Max-2400 X-ray diffractometer with a sealed Cu tube (λ = 1.54178 Å). Thermogravimetric analyses (TGA) were performed at a ramp rate of 10 °C/min in a nitrogen flow with an SDTQ600 instrument. Sample morphologies were recorded *via* scanning electron microscopy (SEM) on a Jeol JSM-6390A field emission scanning electron microscope (FESEM).

^{1}H NMR spectra were recorded on a Varian INOVA-400M type spectrometer with chemical shifts reported as *ppm* (TMS as the internal standard). ^{13}C NMR spectra were measured on a Varian INOVA-500M spectrometer with $CDCl_3$ serving as an internal standard (δ = 77.23). High resolution mass spectra (HRMS) were obtained on a GCT-CA156 Micromass GC/TOF mass spectrometer. GC analysis was performed on an Agilent Technologies 7820A GC system. GC-MS analyses were performed on a Thermo Scientific^{TM} TRACE^{TM} Ultra gas chromatographic instrument.

Solid UV−vis spectra were recorded on an HP 8453 spectrometer. Liquid UV−vis spectra were recorded on a TU-1900 spectrophotometer. Fluorescent spectra and lifetime measurements were recorded on an Edinburgh FS920 fluorescence spectrometer.

HPLC analysis was performed on an Agilent 1100 using a ChIRALPAK AS-H (0.46 cmI.D. x 25 cmL) column purchased from Daicel Chemical Industries, Ltd. Products were purified by flash column chromatography on 200-300 mesh silica gel and Kromasil Lc-80 using a SiO_2 chromatographic column. The CD spectra were measured on a JASCO J-810, by grinding the crystalline sample into powder to be recorded as KBr pellets.

Solid-state cyclic voltammograms were measured on a Zahner PP211 instrument by using a carbon-paste working electrode—a well-ground mixture of each bulk sample and carbon paste (graphite and mineral oil) was set in the channel of a glass tube and connected to a copper wire. A platinum-wire counter electrode and a Ag/AgCl reference electrode were used. Measurements were performed with a three-electrode system in phosphate-buffered saline (PBS) at a scan rate of 50 mV s^{-1}, in the scan range of 0.4−1.2 V.

All confocal laser scanning microscopy (CLSM) micrographs were collected by an Olympus Fluoview FV1000 with λ_{ex} = 488 nm.

Syntheses of InP-1 and **InP-2**

A mixture of **H_3NTB** (37.7 mg, 0.1 mmol), $Zn(NO_3)_2 \cdot 6H_2O$ (29.8 mg, 0.1 mmol) and *L*-**PYI** (15.1 mg, 0.1 mmol) for **InP**-1 or *D*-**PYI** (15.1 mg, 0.1 mmol) for **InP**-2 was dissolved in mixed water (3.0 mL), CH_3OH (1.0 mL) and DMF (2.0 mL). The resulting solution was stirred for approximately 30 min at room temperature, sealed in a 10 mL Teflon-lined stainless steel autoclave, and heated at 110 °C for three days under autogenous pressure. The reaction system was then cooled to room temperature at a rate of 5 °C h^{-1}. Yellow block crystals were collected in 47% yield (based on Zn). Elemental analysis (%) Calc. for $C_{120}H_{125}N_{17}O_{31}Zn_4$: H, 4.92; C, 56.24; N, 9.29. Found: H, 5.12; C, 56.78; N, 8.88 for **InP**-1; Found: H, 4.98; C, 56.13; N, 8.97 for **InP**-2. IR cm^{-1} (KBr): 3467 (br), 2964 (w), 2455 (w), 1676 (m), 1595 (s), 1560 (m), 1504 (m), 1381 (s), 1316 (s), 1278 (m), 1175(m), 1104 (m), 941 (w), 838 (m), 784 (s), 713 (w), 700 (w), 677 (m), 660 (w), 574 (w), 520 (m), 416 (w). ^{1}H NMR (400 MHz, DMSO-d_6/DCl): δ = 9.23 (s, 1H, imidazole ring), 7.85 (d, *J* = 8.4 Hz, 7H, 1H for imidazole ring; 6H for benzene ring), 7.67 (s, 1H, imidazole ring), 7.07 (d, *J* = 8.3 Hz, 6H), 4.66 (ddd, *J* = 18.9, 14.6, 7.0 Hz, 2H), 3.97 (dd, *J* = 12.7, 7.2 Hz, 1H), 3.30 – 3.19 (m, 1H), 3.14 (dd, *J* = 15.9, 9.8 Hz, 1H), 2.14 (td, *J* = 12.0, 7.2 Hz, 1H), 1.97 (dd, *J* = 15.4, 10.4 Hz, 1H), 1.92 – 1.81 (m, 1H), 1.67 (dq, *J* = 17.9, 8.8 Hz, 1H).

Synthesis of InP-3

A mixture of **H_3NTB** (37.7 mg, 0.1 mmol), $Zn(NO_3)_2\cdot6H_2O$ (29.8 mg, 0.1 mmol) was dissolved in mixed water (1.0 mL), pyridine (1.0 mL) and DMF (4.0 mL). The resulting solution was stirred for approximately 30 min at room temperature, sealed in a 10 mL Teflon-lined stainless steel autoclave, and heated at 110 °C for three days under autogenous pressure. The reaction system was then cooled to room temperature at a rate of 5 °C h^{-1}. Light-yellow block crystals were collected in 56% yield (based on Zn). Elemental analysis (%) Calc. for $C_{115}H_{115}N_{13}O_{29}Zn_4$: H, 4.82; C, 57.44.; N, 7.57. Found: H, 4.72; C, 57.89; N, 7.66. IR cm^{-1} (KBr): 3443 (br), 3062 (w), 2961 (w), 2793 (w), 1675 (m), 1624 (s), 1592 (s), 1557 (m), 1505 (m), 1468 (w), 1451 (m), 1373 (s), 1318 (s), 1272 (s), 1217 (w), 1174 (s), 1041 (w), 842 (m), 787 (s), 753 (w), 701 (s), 678 (m), 635 (w), 577(w), 528 (w).

Synthesis of InP-4

Crystals of **InP-**4 were obtained by soaking the crystals of **InP-**1 (0.01 mmol) in a DMF (1.5 mL) solution containing propionaldehyde (0.5 mmol) for 12 h. Elemental analysis (%) Calc. for $C_{125}H_{134}N_{16}O_{33}Zn_4$: H, 5.10; C, 56.65.; N, 8.46. Found: H, 4.93; C, 56.92; N, 8.36. IR cm^{-1} (KBr): 3436 (br), 3135 (w), 2973 (s), 2942 (s), 2880 (s), 1724 (w), 1676 (m), 1624 (s), 1599 (s), 1563 (m), 1507 (w), 1464(m), 1451 (m), 1391 (s), 1374 (s), 1318 (s), 1275 (s), 1242 (m), 1176 (m), 1153 (s), 1106 (m), 1087 (m), 1044 (m), 1027 (m), 1011 (w), 974 (s), 888 (w), 842 (m), 785 (s), 744 (w), 713 (w), 700 (w), 676 (m), 661 (m), 630 (w), 564 (w), 519 (w).

Synthesis of InP-5

Crystals of **InP-**5 were obtained by soaking the crystals of **InP-**2 (0.01 mmol) in a DMF (1.5 mL) solution containing valeraldehyde (0.5 mmol) for 12 h. Elemental analysis (%) Calc. for $C_{128}H_{140}N_{16}O_{32}Zn_4$: H, 5.27; C, 57.45.; N, 8.37. Found: H, 4.98; C, 57.12; N, 8.56. IR cm^{-1} (KBr): 3438 (br), 3135 (w), 2957 (s), 2873 (s), 1710 (m), 1679 (m), 1624 (s), 1596 (s), 1563 (m), 1507 (m), 1464(w), 1451 (m), 1384 (s), 1315 (s), 1278 (s), 1242 (w), 1176 (s), 1146 (s), 1103 (s), 1040 (w), 1014 (w), 991 (m), 964 (m), 944 (m), 845 (m), 786 (s), 742 (w), 716 (w), 703 (w), 680 (m), 663 (m), 633 (w), 574(w), 521 (w).

Typical Procedure for Dye-Uptake of the Catalyst

Before the adsorption of dye, **InP-**1 was first soaked in methanol solution (24 h) for guest molecule exchange and then fully dried in a vacuum oven (120 °C, 12 h) to eliminate the guest molecules. UV-vis measurement of 2′,7′-dichlorofluorescein dye released from dried **InP-**1 (2.56 mg, 2 μmol) and **InP-**1 (2.56 mg, 2 μmol) after catalysis, which were soaked in a methanol solution of 2′,7′-dichlorofluorescein dye (24 mM, 2 mL) in a constant temperature oscillation incubator overnight. The resulting crystals were filtered and washed with methanol thoroughly until the solution became colorless and was then dried under a stream of air. The dried samples were dissociated by concentrated hydrochloric acid, and the resultant clear solution with a light olivine color was diluted to 10 mL and adjusted to a pH of 1.5. The concentration of 2′,7′-dichlorofluorescein dye was determined by comparing the solution UV-vis absorption with a standard curve of the dye.

Typical Procedure for Confocal Laser Scanning Microscopy of the Catalyst

Confocal Laser Scanning Microscopy: The crystals of **InP-**1 were soaked in a methanol solution of 2′,7′-dichorofluorescein dye and conducted using the same procedure with the experiments of dye uptake. The brightfield images and confocal images of the obtained samples were detected at λ_{em}=510–610 nm, exited by λ_{ex}=488 nm through a 405/488 nm filter.

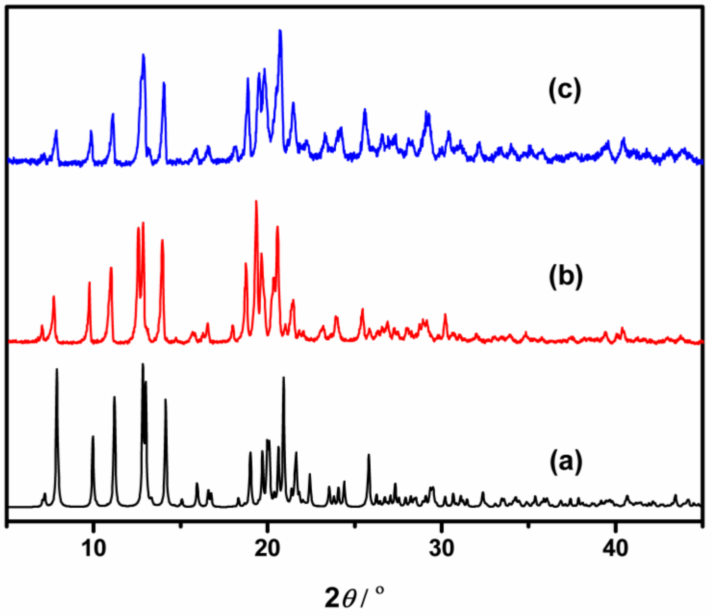

Supplementary Figure 1 │ PXRD patterns of InP-1. The calculated pattern based on the single-crystal simulation (a), the experimental PXRD pattern (b) and the PXRD pattern of the recycled catalyst after three runs of the β-functionalization reaction (c).

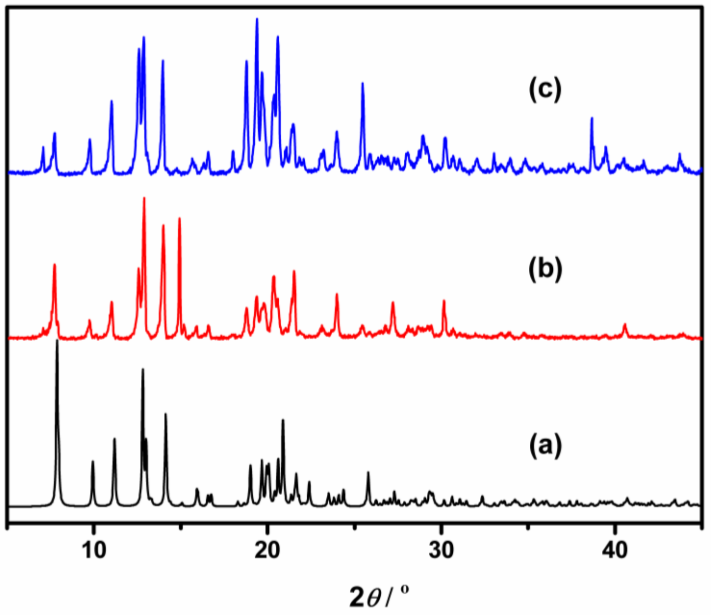

Supplementary Figure 2 │ PXRD patterns of InP-2. The calculated pattern based on the single-crystal simulation (a), the experimental PXRD pattern (b) and the PXRD pattern of the recycled catalyst after three runs of the β-functionalization reaction (c).

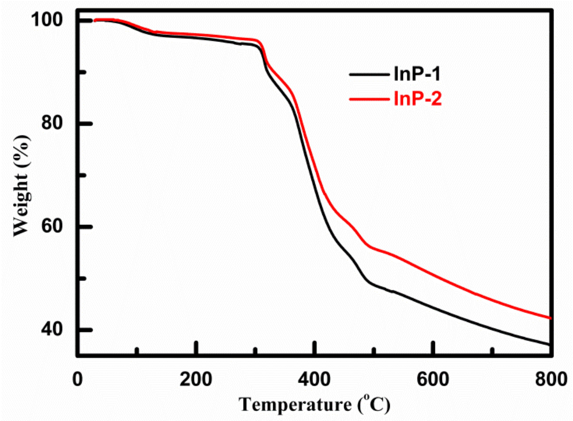

Supplementary Figure 3 │ TG curves of InP-1 (black line) and InP-2 (red line) in the flowing N_2 atmosphere. The skeletons of polymers keep stable before 300 °C.

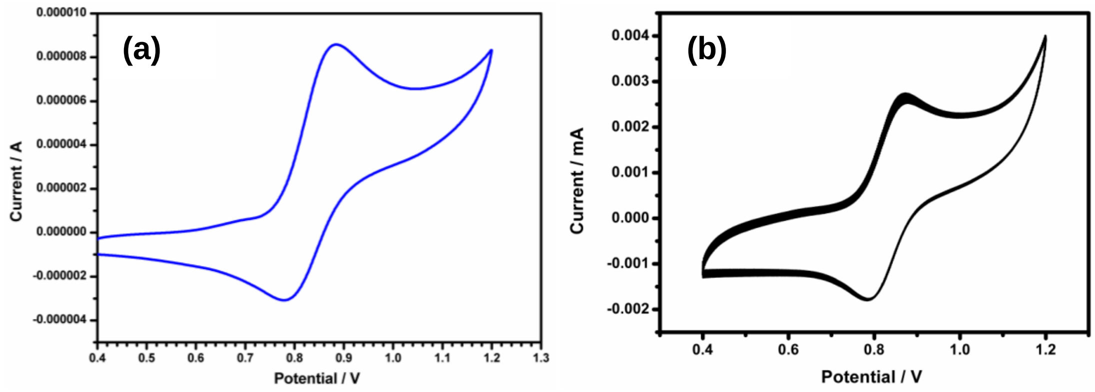

Supplementary Figure 4 │ Solid-state CV of InP-1 (a) and InP-3 (b) with a scan rate of 50 mV s^{-1} in the scan range 0.4−1.2 V.

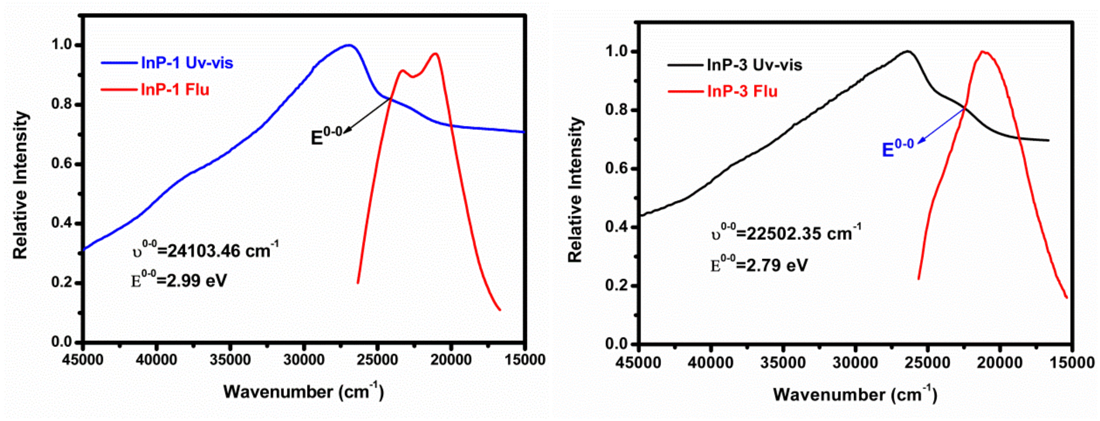

(a) (b)

Supplementary Figure 5 │ Solid-state UV−vis and fluorescence emission spectra of MOF catalysts. (a) normalized (absorption blue line) and emission spectra (red line) of **InP**-1, excited at 369 nm. (b) normalized absorption (black line) and emission spectra (red line) of **InP**-3, excited at 378 nm.

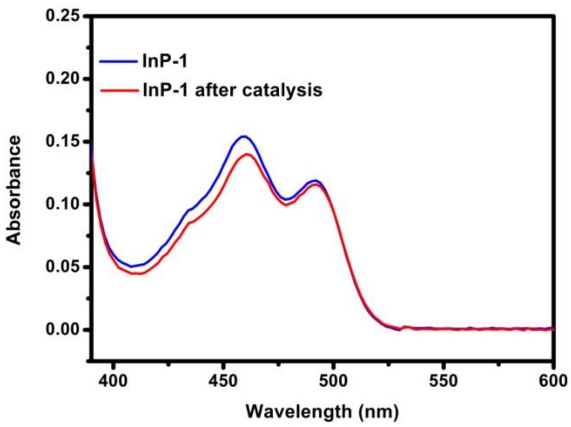

(a)

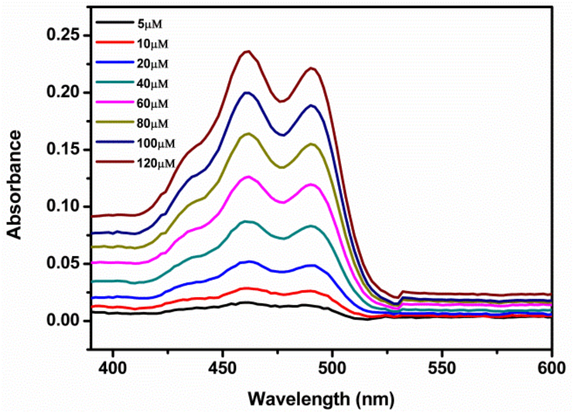

(b)

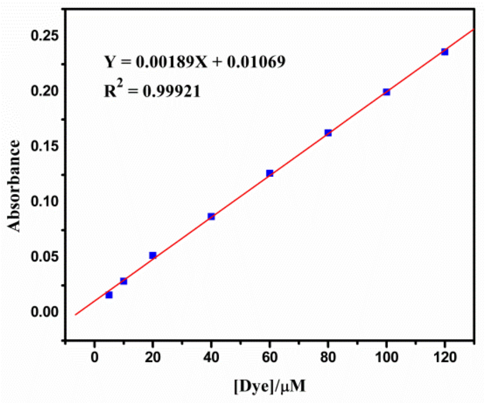

(c)

Supplementary Figure 6 │ UV-vis absorption spectra of 2′,7′-dichlorofluorescein. (a) UV-vis spectra of 2′,7′-dichlorofluorescein dye released from **InP**-1 and **InP**-1 after catalysis. (b) UV-vis spectra of different concentration of 2′,7′-dichlorofluorescein dye in pH = 1.5. (c) The standard linear relationship between the absorption and the dye concentration.

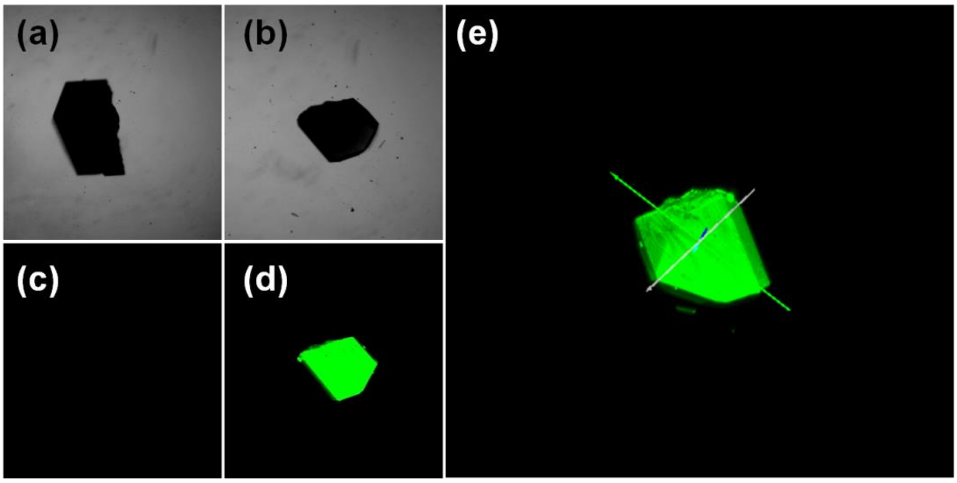

Supplementary Figure 7 │ Confocal images of MOF catalysts. Confocal images of empty (a and c) and soaked (b and d) 2',7'-dichlorofluorescein dye of **InP-**1. Brightfield images (a and b) and confocal images (c and d) detected at λ_{em}=510–610 nm, exited by λ_{ex}=488 nm through a 405/488 nm filter. (e) The 3D reconstruction of **InP-**1 soaked with 2′,7′-dichlorofluorescein dye.

Supplementary Table 1 │ Solid-state CD data of MOF catalysts. CD spectra of three bulky **InP**-1 samples and three bulky **InP**-2 samples with the calculated average values and small standard deviations (σ) of the signals, showing the stability of the CD measurements.

<table>
<tr><th></th><th>Peak 1</th><th>Average 1</th><th>σ</th><th>Peak 2</th><th>Average 2</th><th>σ</th></tr>
<tr><td>InP-1</td><td>266 nm (56.72)</td><td>-</td><td>-</td><td>367 nm (-47.08)</td><td>-</td><td>-</td></tr>
<tr><td>InP-1-first</td><td>262 nm (47.93)</td><td rowspan="3">266 nm
(50.41)</td><td rowspan="3">3.5 nm
(2.17)</td><td>364 nm (-46.94)</td><td rowspan="3">365 nm
(48.07)</td><td rowspan="3">3.6 nm
(2.52)</td></tr>
<tr><td>InP-1-second</td><td>266 nm (51.96)</td><td>362 nm (-46.31)</td></tr>
<tr><td>InP-1-third</td><td>269 nm (51.35)</td><td>369 nm (-50.96)</td></tr>
<tr><td>InP-2</td><td>267 nm (-46.23)</td><td>-</td><td>-</td><td>363 nm (53.09)</td><td>-</td><td>-</td></tr>
<tr><td>InP-2-first</td><td>265 nm (-46.02)</td><td rowspan="3">265 nm
(-45.39)</td><td rowspan="3">4 nm
(5.29)</td><td>357 nm (51.96)</td><td rowspan="3">363 nm
(48.06)</td><td rowspan="3">5.5 nm
(4.51)</td></tr>
<tr><td>InP-2-second</td><td>261 nm (-39.81)</td><td>366 nm (49.82)</td></tr>
<tr><td>InP-2-third</td><td>269 nm (-50.34)</td><td>367 nm (43.31)</td></tr>
</table>

X-ray Crystallography

Summary of crystallographic data and details of data collection for **InP**-1, **InP**-2, **InP**-3, **InP**-4 and **InP**-5 are given in Supplementary Table 2.

Single crystals with suitable dimensions were selected under an optical microscope and mounted onto a glass fiber for data collection. Intensity data for all crystals were collected on a Bruker SMART APEX diffractometer equipped with a CCD area detector and a Mo-Kα (λ = 0.71073 Å) radiation source. The data integration and reduction were processed using SAINT software[5]. An empirical absorption correction was applied to the collected reflections with SADABS[6]. The structures were solved by direct methods using SHELXTL and were refined on F^2 by the full-matrix least-squares method using the SHELXL-97 program[7,8]. All non-hydrogen atoms in the backbone of the polymers were refined anisotropically until convergence was reached. Hydrogen atoms attached to the organic ligands were located geometrically and refined in a riding model, whereas some of the disordered solvent molecules were not treated during the structural refinements. To assist in the stability of the refinement, bond distances between several disordered atoms were fixed. The X-ray crystallographic coordinates for structures reported in this article have been deposited at the Cambridge Crystallographic Data Centre (CCDC) under deposition numbers CCDC 1510628-1510630 and 1540231-1540232. These data can be obtained free of charge from The Cambridge Crystallographic Data Centre *via* www.ccdc.cam.ac.uk/data_request/cif.

Supplementary Table 2 │ Crystal data and refinement parameters for InP-1, InP-2, InP-3, InP-4 and InP-5.

Compound	**InP-1**	**InP-2**	**InP-3**	**InP-4**	**InP-5**
Empirical formula	$C_{120}H_{125}N_{17}O_{31}Zn_4$	$C_{120}H_{125}N_{17}O_{31}Zn_4$	$C_{115}H_{115}N_{13}O_{29}Zn_4$	$C_{125}H_{134}N_{16}O_{33}Zn_4$	$C_{128}H_{140}N_{16}O_{32}Zn_4$
Formula weight	2562.85	2562.85	2404.68	2649.96	2676.04
Crystal system	Monoclinic	Monoclinic	Monoclinic	Monoclinic	Monoclinic
Space group	*C*2	*C*2	*C*2/c	*C*2	*C*2
a/Å	25.016(3)	25.0155(17)	25.9658(15)	24.9168(19)	24.9012(17)
b/Å	9.6759(12)	9.6927(7)	9.0558(5)	9.6298(7)	9.6416(6)
c/Å	27.415(3)	27.3884(18)	27.3690(15)	27.373(3)	27.3223(18)
β/º	116.342(2)	116.2505(11)	117.6864(9)	116.2935(13)	116.3790(10)
V/Å ^{3}	5946.9(13)	5955.9(7)	5698.7(6)	5888.4(9)	5876.7(7)
Z	2	2	2	2	2
D_c/(g·cm^{-3})	1.431	1.429	1.401	1.495	1.512
T (K)	296(2)	200(2)	200(2)	110(2)	110(2)
F(000)	2664	2664	2496	2760	2792
Absorption coefficient/mm^{-1}	0.882	0.881	0.914	0.895	0.897
Reflections collected/unique	19385 / 13424	22473 / 13177	20147 / 6502	21693 / 13000	17779 / 9780
R(int)	0.0304	0.0188	0.0279	0.0306	0.0241
Data/restraints/parameters	13424 / 26 / 778	13177 / 26 / 778	6502 / 44 / 391	13000 / 39 / 785	9780 / 89 / 801
Goodness-of-fit on F^2	1.088	1.064	1.033	1.009	1.054
R_1[a] [I>2σ(I)]	0.0592	0.0491	0.0513	0.0457	0.0406
wR_2[b] [I>2σ(I)]	0.1651	0.1726	0.2017	0.1462	0.1182
R_1[a] (all data)	0.0809	0.0551	0.0627	0.0495	0.0429
wR_2[b] (all data)	0.1802	0.1792	0.2161	0.1508	0.1198
Flack parameter	0.047(17)	0.070(13)	—	0.070(10)	0.048(14)
CCDC number	1510628	1510629	1510630	1540232	1540231

[a] $R_1 = \sum||F_o| - |F_c||/\sum|F_o|$

[b] $wR_2 = [\sum w(F_o^2 - F_c^2)^2/\sum w(F_o^2)^2]^{1/2}$

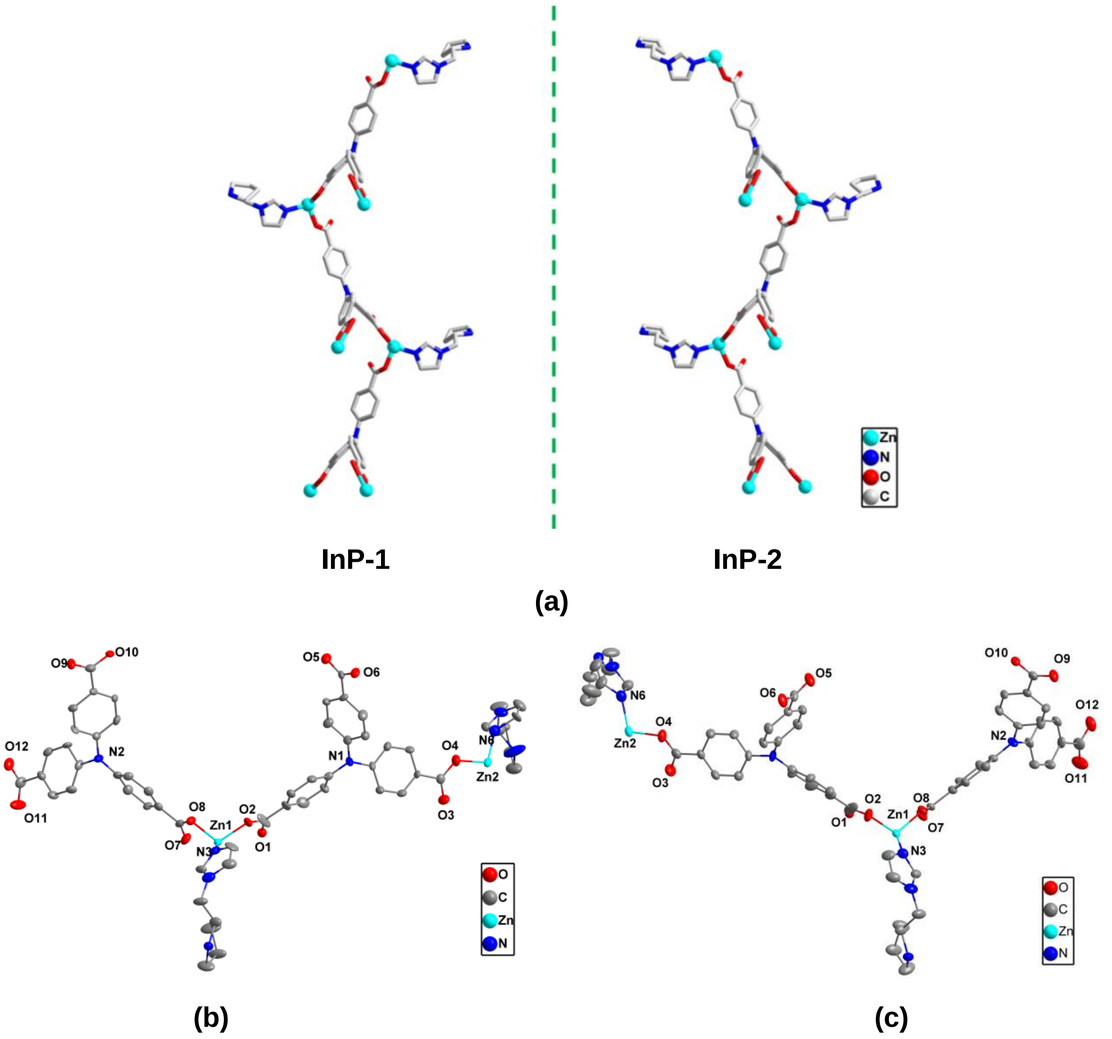

Supplementary Figure 8 │ Crystal structures of InP-1 and InP-2. (a) Mirror-image structures of **InP**-1 and **InP**-2. Ellipsoid diagram (50% probability) of **InP**-1 (b) and **InP**-2 (c) in an asymmetric unit with selected labelling scheme. Free solvent molecules and all H atoms are omitted for clarity.

Selective bond distance(Å) in **InP**-1: Zn(1)-O(2) 1.955(3), Zn(1)-O(8) 1.956(2), Zn(1)-N(3) 2.003(3), Zn(1)-O(10) 2.011(3), Zn(2)-O(6) 1.929(3), Zn(2)-O(4) 1.946(2), Zn(2)-O(12) 1.994(3), Zn(2)-N(6) 2.051(4). Selected bond angle (º): O(2)-Zn(1)-O(8) 109.0(2), O(2)-Zn(1)-N(3) 95.8(2), O(8)-Zn(1)-N(3) 112.6(2), O(2)-Zn(1)-O(10) 110.2(2), O(8)-Zn(1)-O(10) 117.8(2), N(3)-Zn(1)-O(10) 109.2(2), O(6)-Zn(2)-O(4) 116.6(2), O(6)-Zn(2)-O(12) 110.5(2), O(4)-Zn(2)-O(12) 109.4(2), O(6)-Zn(2)-N(6) 110.7(2), O(4)-Zn(2)-N(6) 111.4(2), O(12)-Zn(2)-N(6) 96.5(2).

Selective bond distance(Å) in **InP**-2: Zn(1)-O(8) 1.952(2), Zn(1)-O(2) 1.961(2), Zn(1)-O(10) 1.984(2), Zn(1)-N(3) 2.025(2), Zn(2)-O(6) 1.947(2), Zn(2)-O(4) 1.948(2), Zn(2)-O(12) 1.988(2), Zn(2)-N(6) 2.018(3). Selected bond angle: O(8)-Zn(1)-O(2) 108.7(9), O(8)-Zn(1)-O(10) 118.2(1), O(2)-Zn(1)-O(10) 110.6(1), O(8)-Zn(1)-N(3) 112.1(1), O(2)-Zn(1)-N(3) 96.1(2), O(10)-Zn(1)-N(3) 109.0(1), O(6)-Zn(2)-O(4) 116.9(1), O(6)-Zn(2)-O(12) 110.0(1), O(4)-Zn(2)-O(12) 109.5(1), O(6)-Zn(2)-N(6) 109.7(2), O(4)-Zn(2)-N(6) 112.0(1), O(12)-Zn(2)-N(6) 96. 9(2).

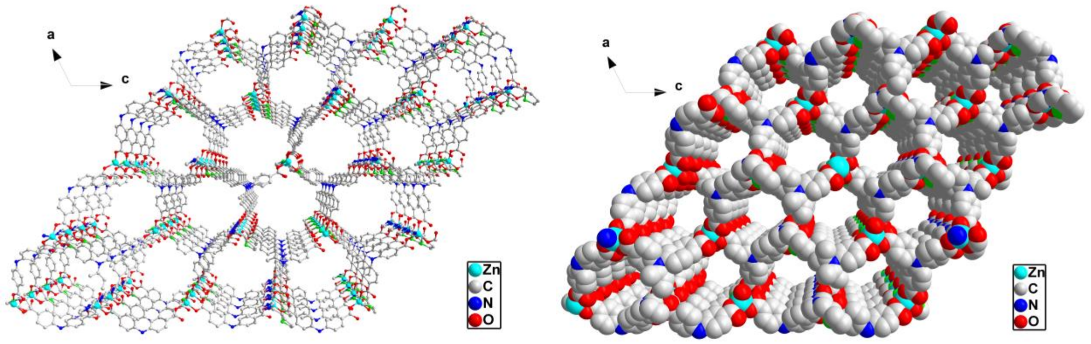

(a) (b)

Supplementary Figure 9 │ 3D structure of the isolated InP-1 before interpenetration viewed along the *b*-axis. Ball-Stick mode (a) and Spacefill mode (b).

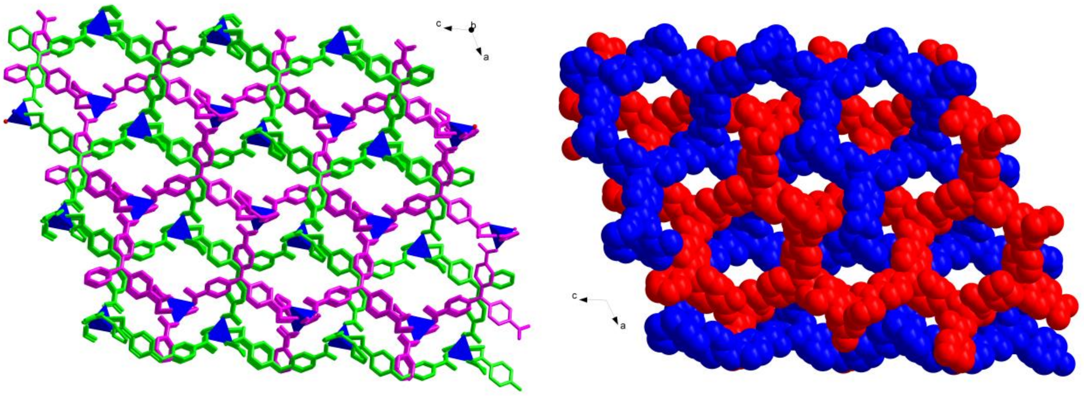

(a) (b)

Supplementary Figure 10 │ Two-fold interpenetrated net of InP-1 viewed along the *b*-axis. Ball-Stick mode (a) and Spacefill mode (b).

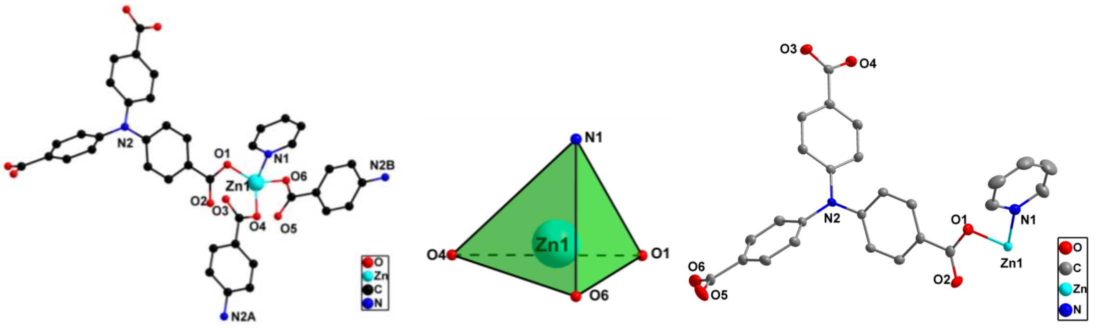

(a) (b) (c)

Supplementary Figure 11 │ Crystal structures of InP-3. The coordination environment (a) and the distorted tetrahedral coordination geometry (b) of Zn(II) ion in **InP**-3. (c) Ellipsoid diagram (50% probability) of **InP**-3 in an asymmetric unit with selected labelling scheme. Free solvent molecules, anion and all H atoms are omitted for clarity. Selective bond distance(Å) in **InP**-3: Zn(1)-O(1) 1.948(2), Zn(1)-O(4) 1.953(2), Zn(1)-O(6) 1.960(2), Zn(1)-N(1) 2.087(2). Selected bond angle (º): O(1)-Zn(1)-O(4) 130.1(6), O(1)-Zn(1)-O(6) 107.5(7), O(4)-Zn(1)-O(6) 107.5(7), O(1)-Zn(1)-N(1) 100.7(7), O(4)-Zn(1)- N(1) 109.7(7), O(6)-Zn(1)-N(1) 96.1(7).

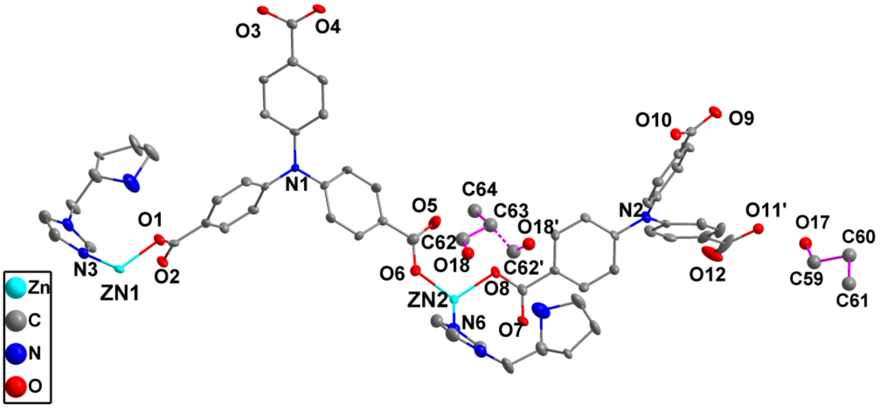

(a)

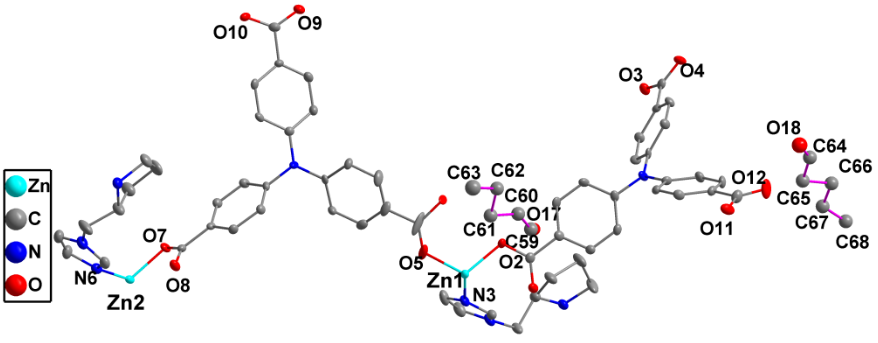

(b)

Supplementary Figure 12 │ Ellipsoid diagram (50% probability) of InP-4 (a) and InP-5 (b) in an asymmetric unit with selected labeling scheme. Free solvent molecules and all H atoms are omitted for clarity.

General Procedure for the *β*-Arylation of Saturated Aldehydes

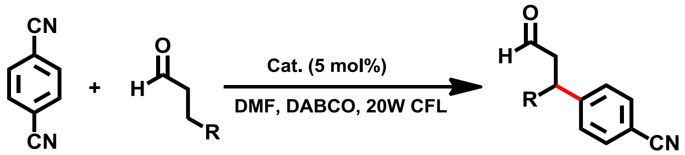

A glass tube was filled with 1,4-dicyanobenzene (1.0 mmol, 1.0 equiv), **InP**-1 (0.025 mmol, 0.025 equiv) and 1,4-diazabicyclo[2.2.2]octane (DABCO, 5.0 mmol, 5.0 equiv). Then, this vial was purged with N_2, and the corresponding aldehyde (1.4 mmol, 1.4 equiv) and DMF (3.0 mL) were added *via* syringe. The resulting mixture was then cooled to –78 °C and degassed *via* vacuum evacuation, backfilled with N_2, and warmed to room temperature. This process was repeated three times. With all the steps complete, the vial was placed approximately 5 cm away from a 20 W fluorescent lamp source for 48 h of uninterrupted irradiation. After reaction, the mixture was centrifuged to remove **InP**-1 catalyst, which was washed with DMF and MeOH for standby. The conversions were calculated from GC analyses of the supernatant solution using internal standards. The reaction mixture was extracted with ethyl acetate, dried with anhydrous Na_2SO_4, concentrated in vacuo and purified by flash chromatography on silica gel using hexane/ethyl acetate as the eluent to give the *β*-arylated aldehyde product as an oil. For the aromatic aldehyde substrates, the supernatant solution after centrifugation was diluted with 8 mL of DCM and 2 mL of MeOH. The reaction mixture was then cooled to 0 °C and sodium borohydride (5.0 mmol, 5.0 equiv) was added to reduce the resulting aldehyde to the corresponding alcohol for GC analysis and isolation. Then, the reaction mixture was extracted with ethyl acetate, dried with anhydrous Na_2SO_4, and concentrated in vacuo. The crude product was purified by column chromatography over silica gel using hexane/ethyl acetate as the eluent to afford the *β*-arylated product as an oil.

Supplementary Table 3 │ Photocatalytic *β*-arylation of aldehydes with InP-1/InP-2 as catalysts.[a]

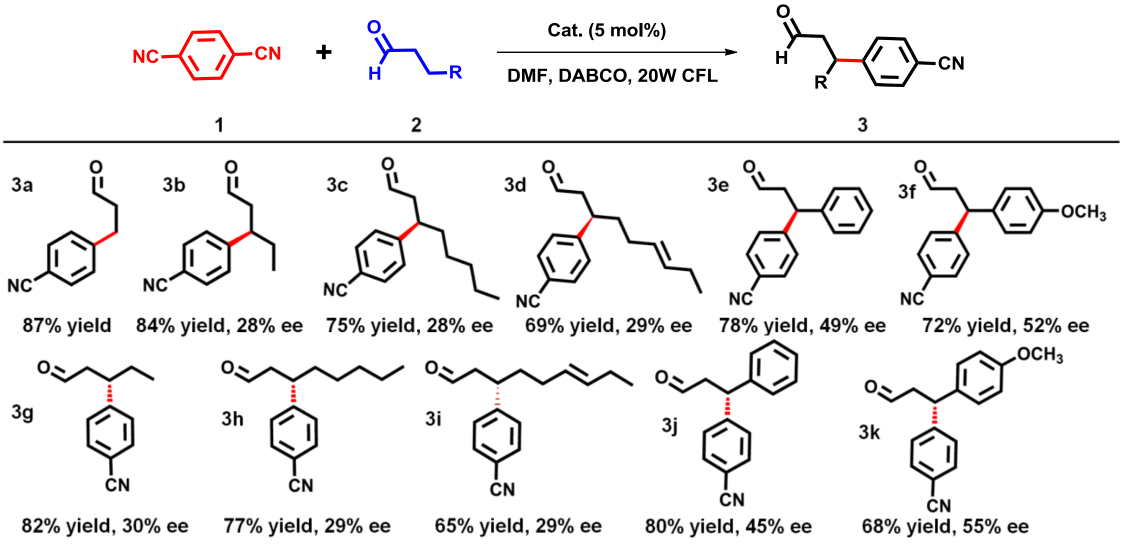

[a] Reaction conditions: 1,4-dicyanobenzene (1.0 mmol), aldehydes (1.4 mmol), **InP**-1/**InP**-2 (5 mol%, based on **H_3NTB**), DABCO (5 mmol), DMF (3 mL). The conversions were determined by GC analysis using biphenyl as an internal standard. The *ee* values were determined by HPLC analysis.

Supplementary Table 4 │ Control catalytic experiments of *β*-arylation of propionaldehyde.[a]

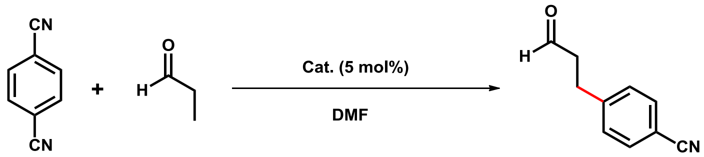

Entry	Catalyst	Cocatalyst(equiv)	Conversion[b]
1	**H_3NTB**	DABCO(5)	—
2	*L*-**PYI**	DABCO(5)	—
3	—	DABCO(5)	—
4	—	—	—
5	**InP**-1	DABCO(5)	trace [c]
6 [d]	—	DABCO(5)+HOAc(0.2) +H_2O(3)+DMPU(2 mL)	—
7	$Zn(NO_3)_2{\cdot}6H_2O$	DABCO(5)	—
8	**H_3NTB**+*L*-**PYI**	DABCO(5)	11%
9	$Zn(NO_3)_2{\cdot}6H_2O$+**H_3NTB**+*L*-**PYI**	DABCO(5)	26%
10	**InP**-3	DABCO(5)	—
11	**InP**-3+*L*-**PYI**	DABCO(5)	58%
12	MOF-**150**	DABCO(5)	—
13	MOF-**150**+*L*-**PYI**	DABCO(5)	54%
14	Zn-PYI**1**	DABCO(5)	52%

[a] Reaction conditions: 1,4-dicyanobenzene (1.0 mmol), propionaldehyde (1.4 mmol), cat. (5 mol%, based on **H_3NTB**), DMF (3 mL), in N_2, fluorescent lamp (20 W), 48 h. [b] Determined by GC analysis with internal standard. [c] In dark. [d] Simulating the conditions in the reported homogeneous system with free catalysts[9].

Supplementary Table 5 │ Recycle catalytic experiments of *β*-arylation of propionaldehyde catalyzed by InP-1 and InP-2 (in parentheses) in the optimum conditions.

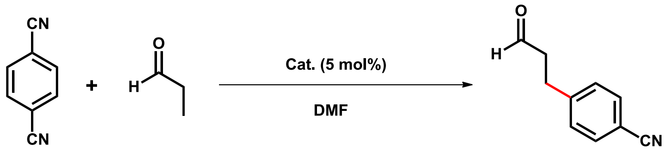

Round	Time (h)	Conversion/%
1	48	87(84)
2	48	85(80)
3	48	82(79)

Supplementary Table 6 │ Conversion vs. time for the *β*-arylation reaction. Catalytic traces of the *β*-arylation reaction performed by **InP**-1, **InP**-3 with 5 mol% *L*-**PYI** and **Zn-PYI**1 under the same conditions with different reaction time.[a]

Conversion (%) / Time (h)	**InP**-1	**InP**-3+*L*-**PYI**	**Zn-PYI**1	**InP**-1[b]
3	8.73	15.9	11.4	10.6
6	25.1	26.7	19.7	30.4
9	36.8	35.3	25.4	42.1
12	44.1	39.6	31.2	51.7
24	68.4	48.7	42.5	66.5
30	77.2	52.4	46.0	74.3
36	84.1	55.1	48.7	82.5
48	87.4	58.6	52.6	84.8

[a] Reaction conditions: 1,4-dicyanobenzene (1.0 mmol), propionaldehyde (1.4 mmol), catalyst (0.05 mmol), DABCO (5.0 mmol), 20 W fluorescent lamp, 3 mL DMF, in N_2. [b] The addition amount of propionaldehyde increased to 2.0 mmol. The conversions were determined by GC analysis.

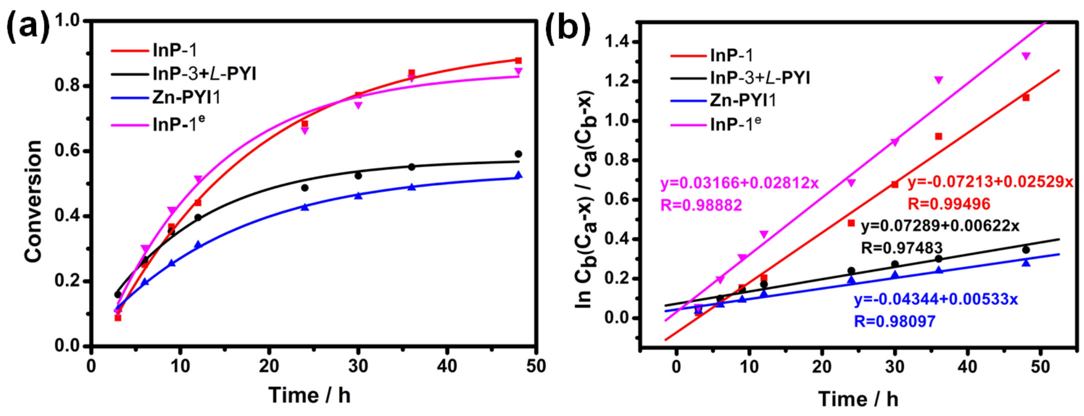

Supplementary Figure 13 │ Reaction kinetics experimental results. Time-dependent conversions (a) and kinetic plots (b) of the *β*-arylation of propionaldehyde catalyzed by **InP**-1, **InP**-3 with 5 mol% *L*-**PYI** and **Zn-PYI**1. C_a and C_b are the initial concentration of 1,4-dicyanobenzene (0.333 M), propionaldehyde (0.467 M), respectively; x is the concentration consumption; e represents that the initial concentration C_b increases to 0.667 M.

Supplementary Table 7 │ Control experiments of *β*-arylation of valeraldehyde.[a]

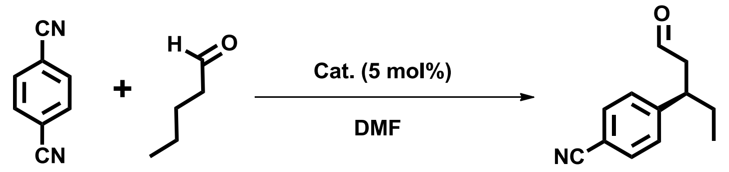

Entry	Catalyst	Conversion [b]	ee
1	**H_3NTB**+*L*-**PYI**	9%	–
2	MOF-**150**	trace	–
3	MOF-**150** +5 mol% *L*-**PYI**	52%	–
4	MOF-**150** +20 mol% *L*-**PYI**	58%	–
5	Zn-**PYI**1	44%	<1%
6	**InP**-3+5 mol% *L*-**PYI**	47%	–

[a] Reaction conditions: 1,4-dicyanobenzene (1.0 mmol), valeraldehyde (1.4 mmol), cat. (5 mol%), DABCO (5.0 mmol), DMF (3 mL), in N_2, fluorescent lamp (20 W), 48 h. [b] Determined by GC analysis with internal standard.

Synthesis of bulky aldehyde (E)-3-(3-oxoprop-1-enyl)phenyl 3,5-di-tert-butylbenzoate[10]

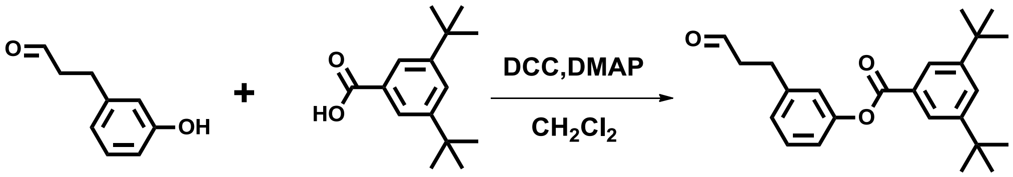

To a CH_2Cl_2 solution of 3,5-di-*tert*-butylbenzoic acid (0.234 g, 1.0 mmol) was added DCC (0.228 g, 1.05 mmol), DMAP (0.122 g, 1.0 mmol) under 0 °C and stirred for 1 h. 3-(3-hydroxyphenyl)propane (0.15 g, 1.0 mmol) in 5 mL of CH_2Cl_2 was dropwise added and the mixture was stirred for another 12 h. The reaction mixture was filtered through a pad of Celite and washed with CH_2Cl_2. The combined solutions were washed with 5% $NaHCO_3$, brine, dried over anhydrous Na_2SO_4, and concentrated. The crude product was purified by column chromatography over silica gel (EA:Hexane = 1:9) to afford the bulky aldehyde (Yield: 55%) as yellow oil. ^{1}H NMR (400 MHz, $CDCl_3$) δ = 9.74 (t, 1H), 7.42-7.39 (m, 1H), 7.28-7.23 (m, 1H), 7.11-7.07 (m, 4H), 6.96-6.94 (m, 1H), 2.88-2.84 (t, 2H), 2.75-2.71 (t, 2H), 1.20-1.18 (m, 18H).

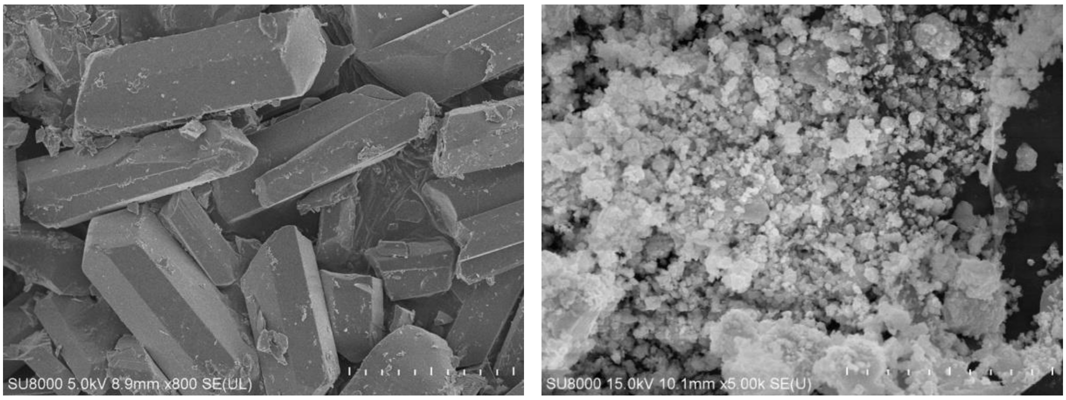

(a) (b)

Supplementary Figure 14 │ SEM images of InP-1. (a) SEM image (scale bar: 50 μm) of the initial **InP-1** crystals with size of 50~80 μm, with which the conversion of the reaction between 1,4-dicyanobenzene and benzenepropanal under the optimal condition is 52% after 24 h of irradiation. (b) SEM image (scale bar: 10 μm) of the grinded **InP-1** sample with size of 0.1~1 μm, with which a comparable conversion of 56% under the same conditions was detected.

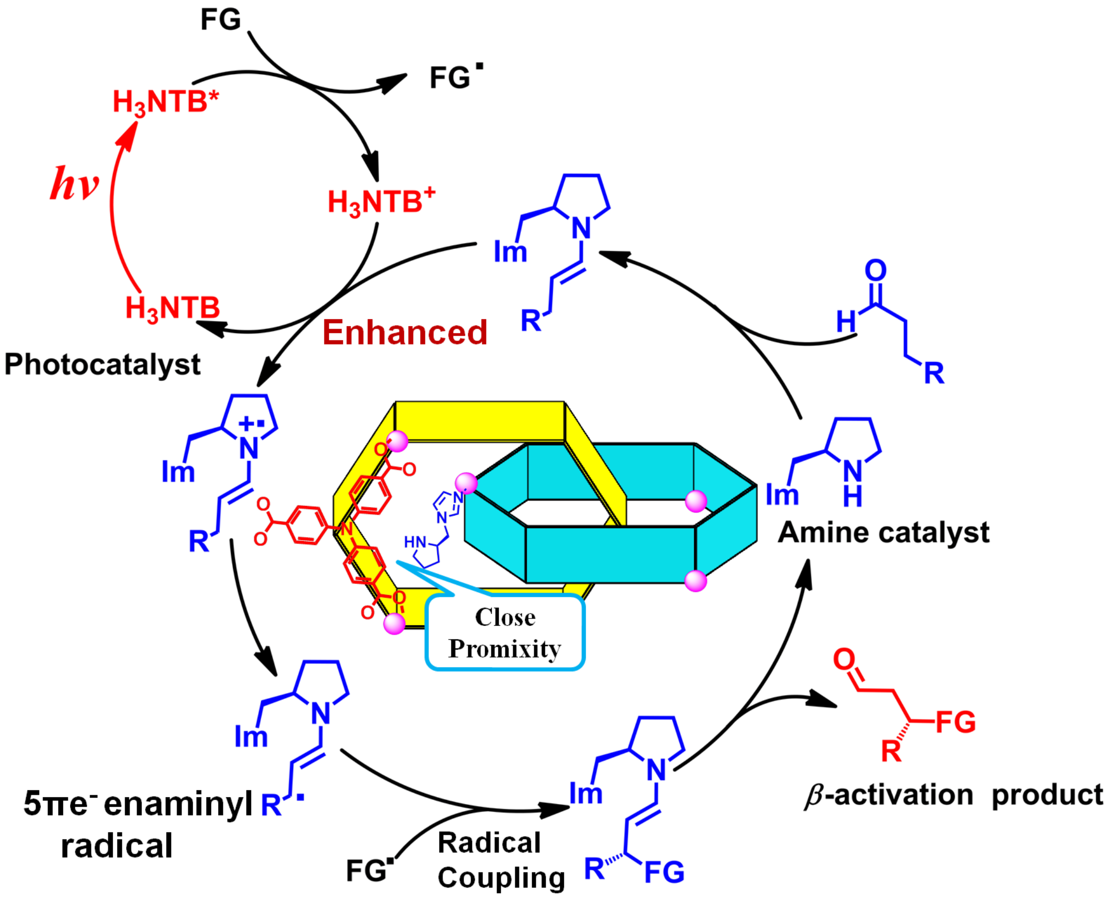

Supplementary Figure 15 │ Proposed photocatalytic reaction mechanism. The proposed mechanistic pathway of the direct photocatalytic *β*-functionalization of saturated aldehydes with the interpenetrated MOF catalysts. R, generic organic substituent; FG, functional group; Im, imidazole.

General Procedure for the *β*-Functionalization of Saturated Ketones

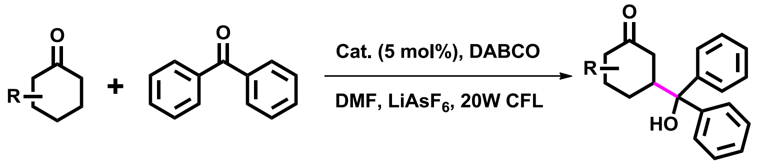

A glass tube was filled with benzophenone (0.5 mmol, 1.0 equiv), **InP**-1 (12.5 μmol, 0.025 equiv), $LiAsF_6$ (0.5 mmol, 1.0 equiv) and 1,4-diazabicyclo[2.2.2]octane (DABCO, 1.0 mmol, 2.0 equiv). Then, this vial was purged with N_2, and the corresponding cyclohexanone (2.5 mmol, 5.0 equiv) and DMF (2.0 mL) was added *via* syringe. The resulting mixture was then cooled to –78 °C and degassed *via* vacuum evacuation, backfilled with N_2, and warmed to room temperature. This process was repeated three times. With all the steps complete, the vial was placed approximately 5 cm away from a 20 W fluorescent lamp source for 48 h of uninterrupted irradiation. After reaction, the mixture was centrifuged to remove **InP**-1 catalyst, which was washed with DMF and ethanol for standby. The conversions were calculated from GC-MS analyses of the supernatant solution. The reaction mixture was diluted with water and extracted with ethyl acetate. The combined organic layers were washed with brine, dried with anhydrous Na_2SO_4, concentrated in vacuo and purified by flash chromatography on silica gel using hexane/ethyl acetate as the eluent to provide an inseparable mixture of the desired *β*-adduct and the corresponding hemiacetal.

Supplementary Table 8 │ Photocatalytic *β*-functionalization of ketones with InP-1 as catalyst.[a]

Entry	Substrate	Product	Conversion/%[b]
1	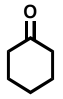	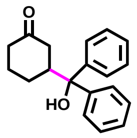	67
2	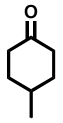	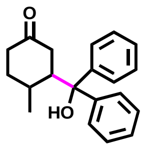	62
3	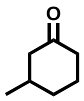	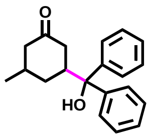	55
4	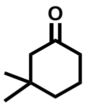	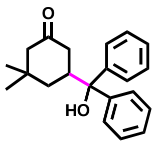	38
5	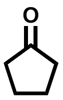	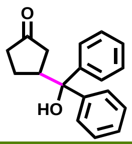	47

[a] Reaction conditions: benzophenone (0.5 mmol), ketones (2.5 mmol), **InP**-1 (5 mol%, based on **H_3NTB**), $LiAsF_6$ (0.5 mmol), DABCO (1.0 mmol), DMF (2 mL), N_2, 20-watt fluorescent lamp with illumination distance of 5 cm, 48 h. [b] The conversions were determined by GC-MS analysis.

Host-Guest Properties between the Catalysts and Substrates

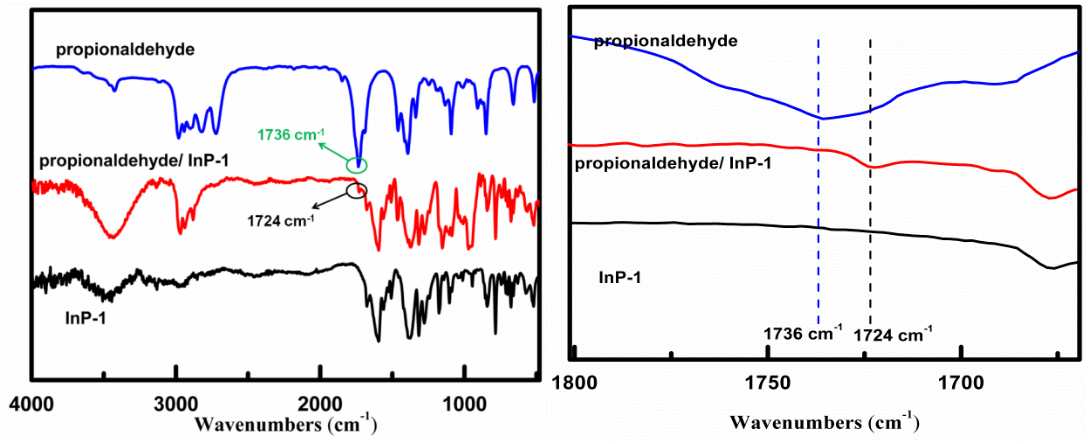

Supplementary Figure 16 │ IR spectra of propionaldehyde (blue line), InP-1 (black line), and InP-1 with absorbed propionaldehyde (red line). The redshift of the characteristic signals of $v^{st}{}_{C=O}$ from 1736 cm^{-1} (free propionaldehyde) to 1724 cm^{-1} shows the adsorption and activation of substrate in the **InP**-1.

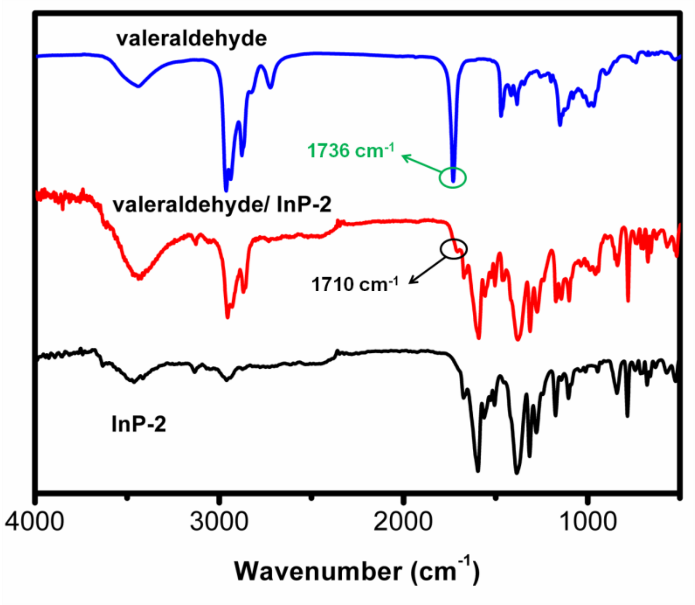

Supplementary Figure 17 │ IR spectra of valeraldehyde (blue line), InP-2 (black line), and InP-2 with absorbed valeraldehyde (red line). The redshift of the characteristic signals of $v^{st}{}_{C=O}$ from 1736 cm^{-1} (free propionaldehyde) to 1710 cm^{-1} shows the adsorption and activation of substrate in the **InP**-2.

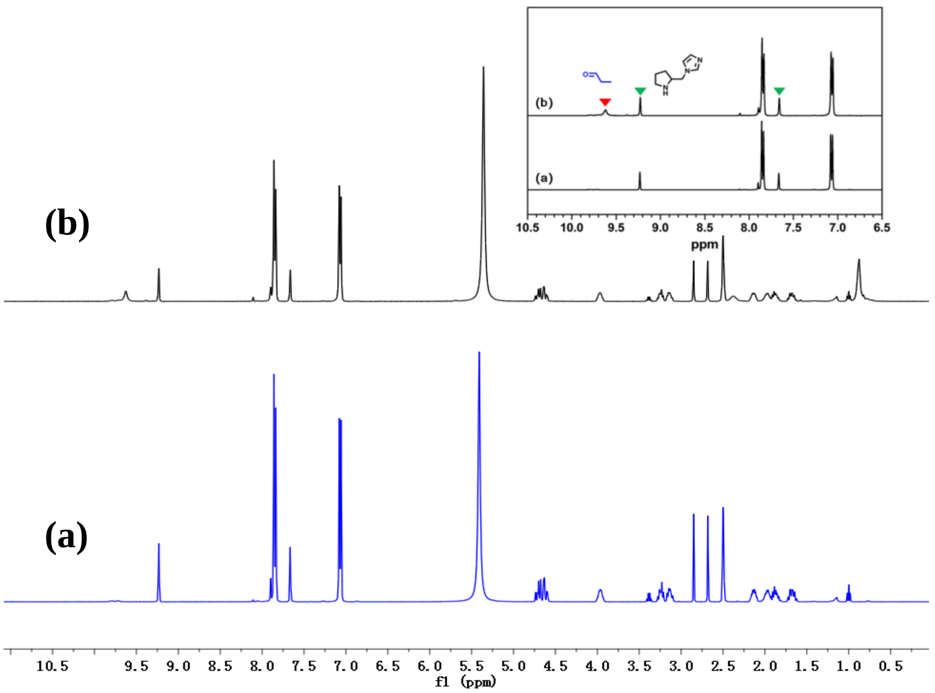

Supplementary Figure 18 | ^{1}H NMR spectra of InP-1 (a) and InP-1 with adsorbed propionaldehyde (b) dissolved in DMSO-d_6/DCl. Peaks marked with red and green triangles represent the signals of propionaldehyde and *L*-**PYI**, respectively.

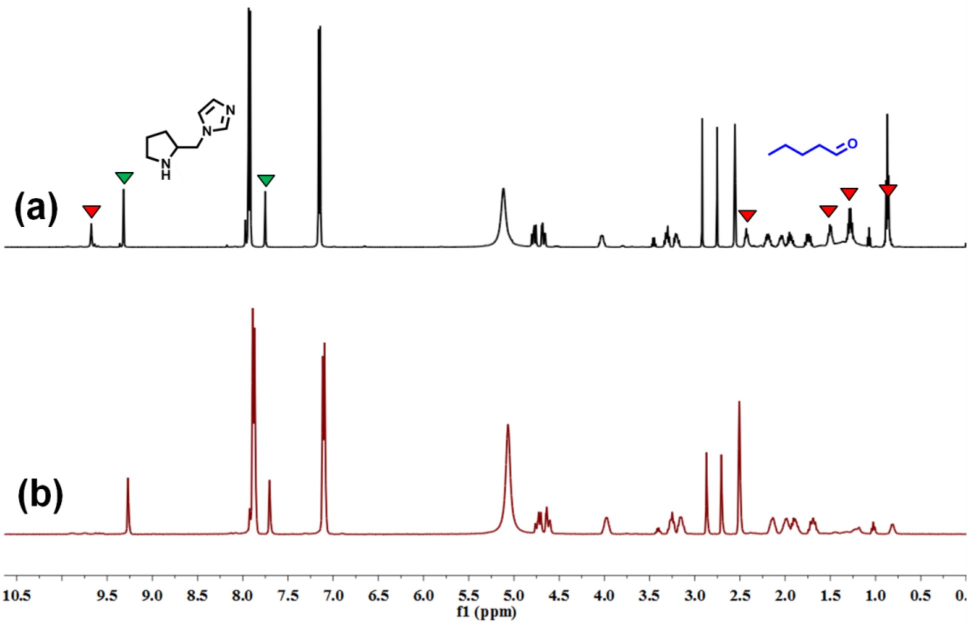

Supplementary Figure 19 | ^{1}H NMR spectra of InP-2 (a) and InP-2 with adsorbed valeraldehyde (b) dissolved in DMSO-d_6/DCl. Peaks marked with red and green triangles represent the signals of valeraldehyde and *D*-**PYI**, respectively.

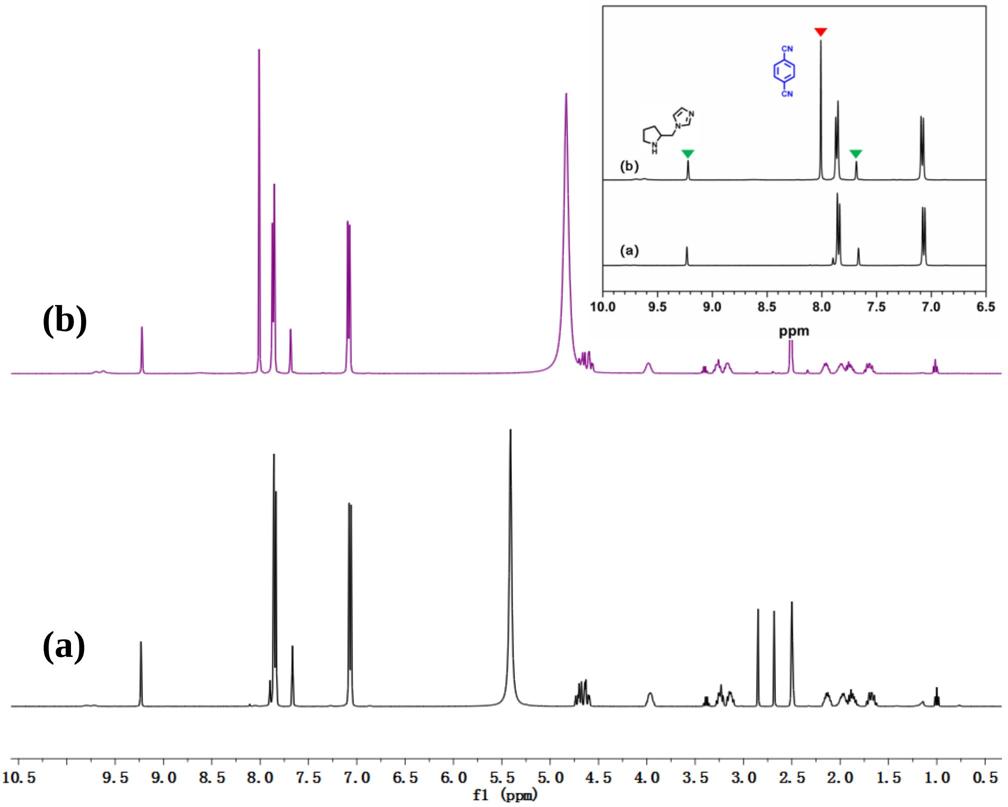

Supplementary Figure 20 | ^{1}H NMR spectra of InP-1 (a) and InP-1 with adsorbed 1,4-dicyanobenzene (b) dissolved in DMSO-d_6/DCl. Peaks marked with red and green triangles represent the signals of 1,4-dicyanobenzene and *L*-**PYI**, respectively.

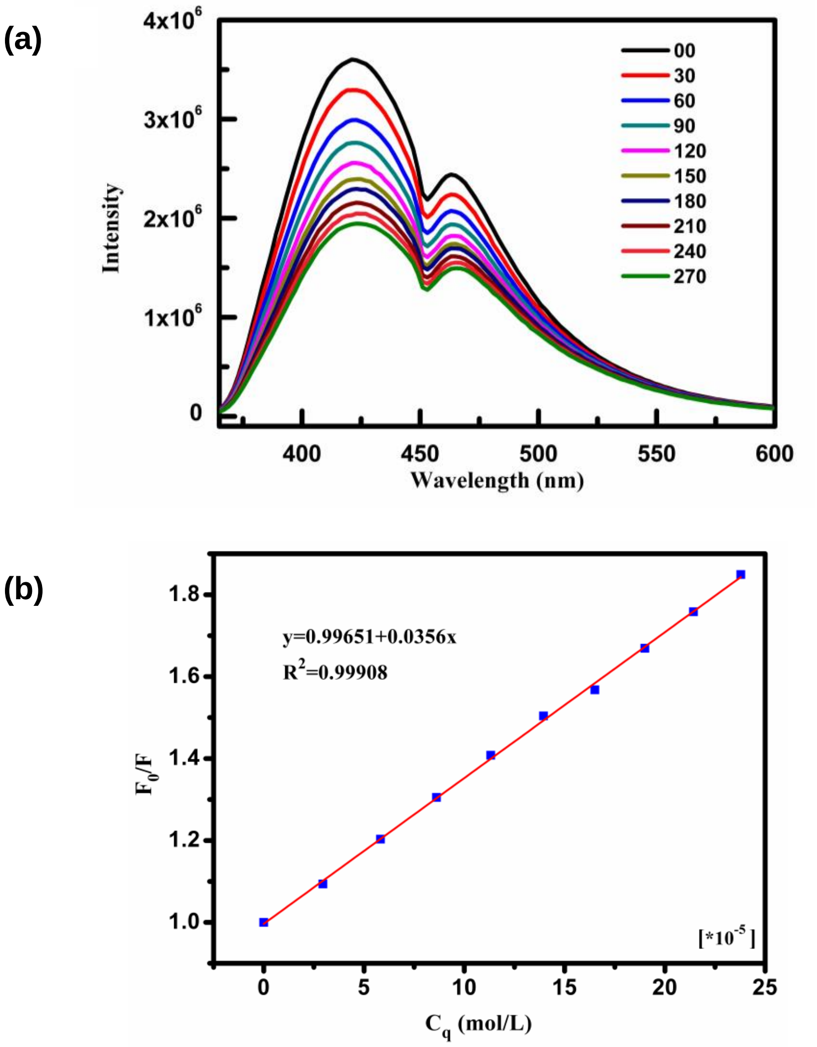

Supplementary Figure 21 │ Fluorescence quenching spectra of InP-1 **upon the addition of 1,4-dicyanobenzene** (a) and the corresponding simulated Stern-Volmer curve (b).

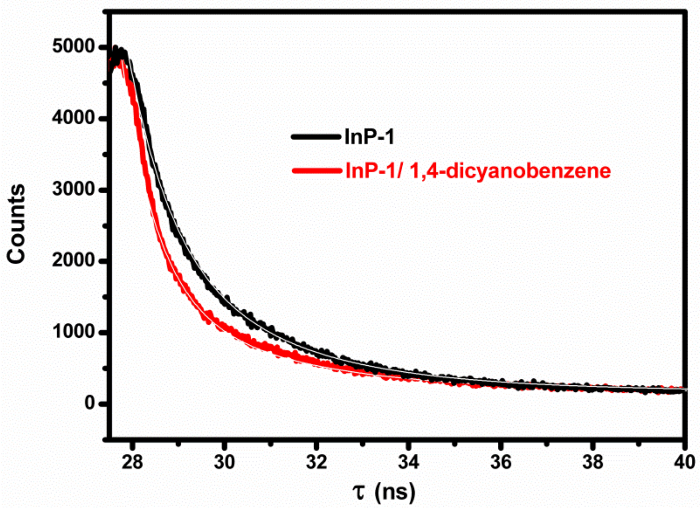

Supplementary Figure 22 │ Fluorescence decay curve of InP-1 suspension (black line) and of the **InP-**1 suspension upon addition of 1,4-dicyanobenzene (50 μM) (red line). The intensity was recorded at 440 nm using 405.2 nm laser as resource.

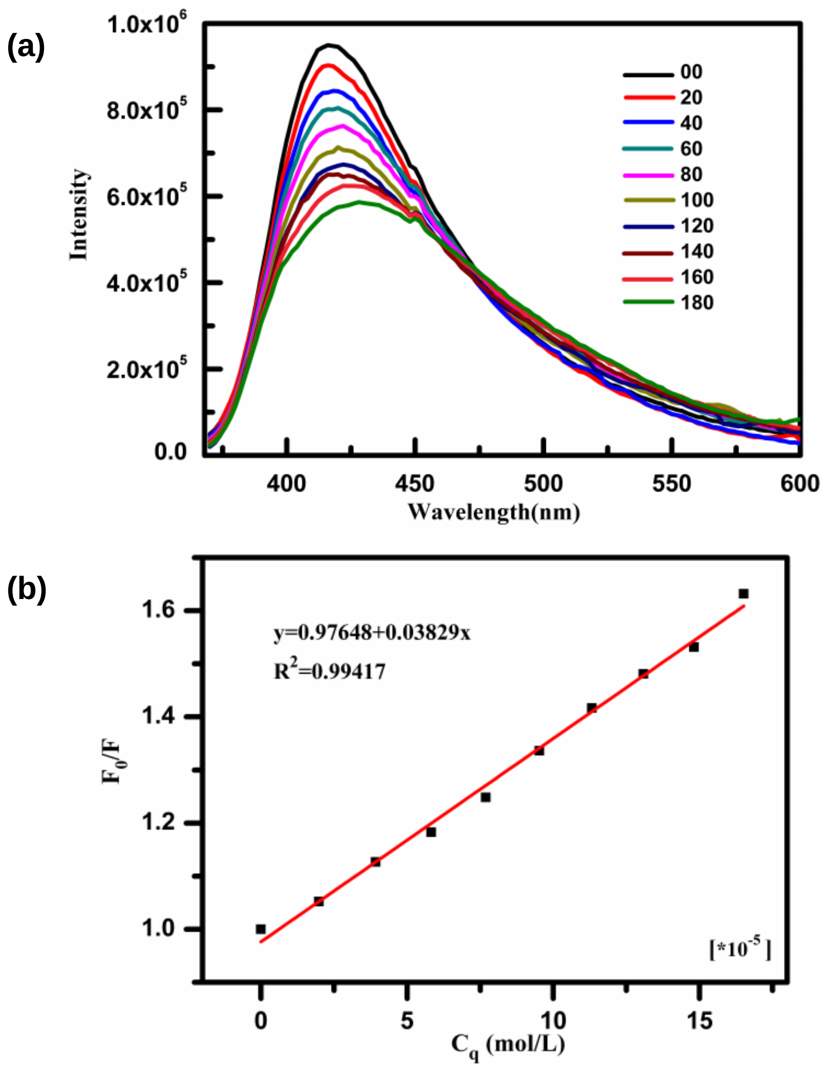

Supplementary Figure 23 │ Fluorescence quenching spectra of InP-3 upon the addition of 1,4-dicyanobenzene (a) and the corresponding simulated Stern-Volmer curve (b).

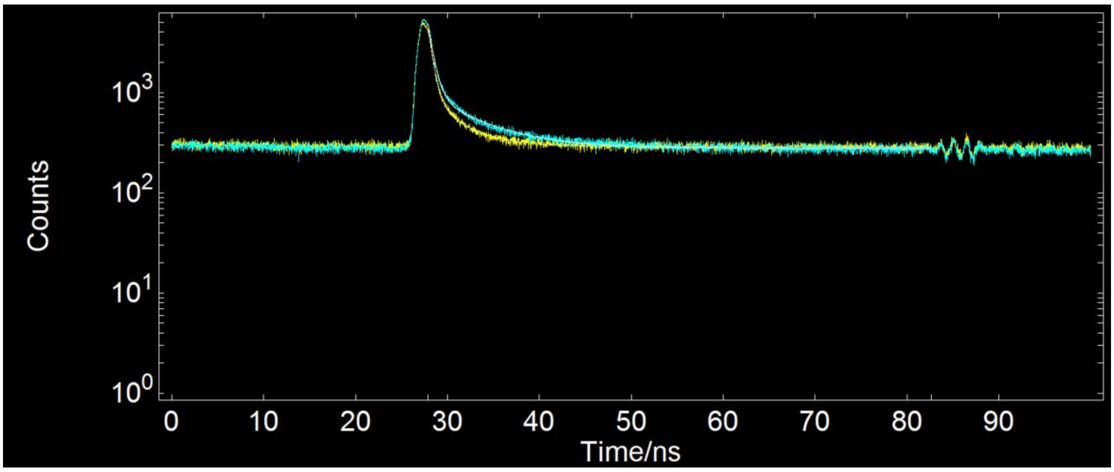

Supplementary Figure 24 │ Fluorescence decay curve of InP-3 suspension (turquoise line) and of the **InP**-3 suspension upon addition of 1,4-dicyanobenzene (50 μM) (yellow line). The intensity was recorded at 426 nm using 405.2 nm laser as resource.

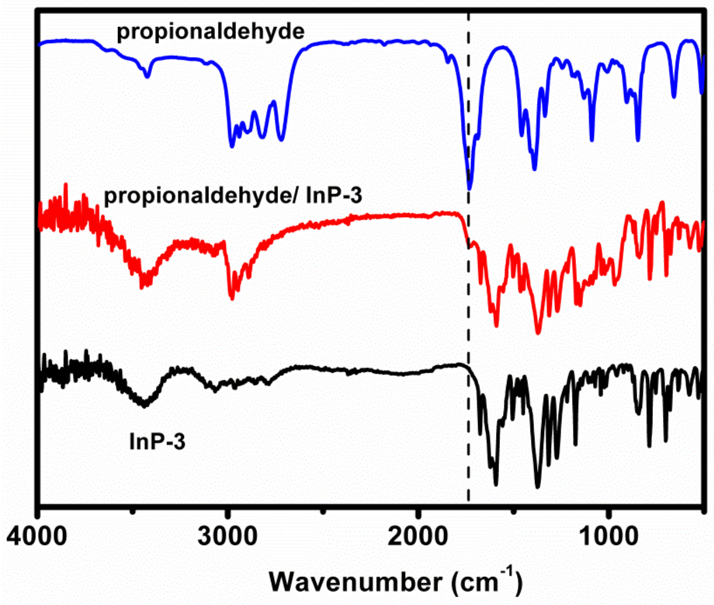

Supplementary Figure 25 │ IR spectra of propionaldehyde (blue line), **InP**-3 (black line), and **InP**-3 with absorbed propionaldehyde (red line).

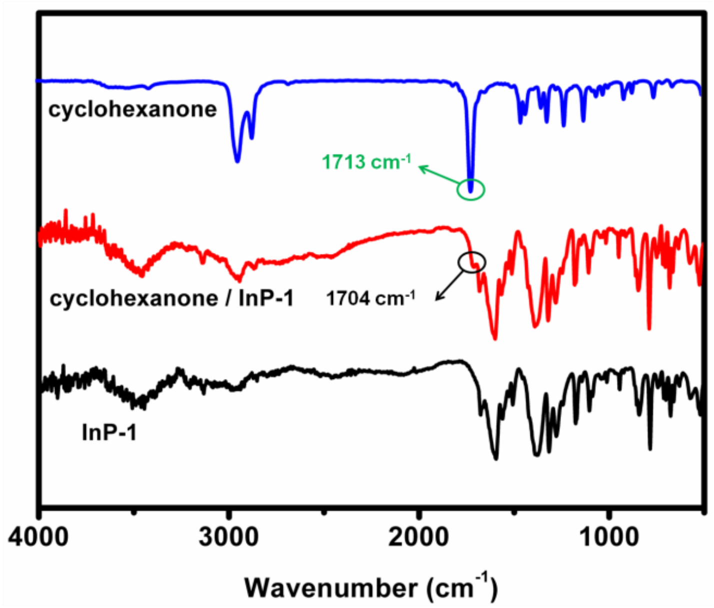

Supplementary Figure 26 │ IR spectra of cyclohexanone (blue line), **InP**-1 (black line), and **InP**-1 with absorbed cyclohexanone (red line).

(a)

(b)

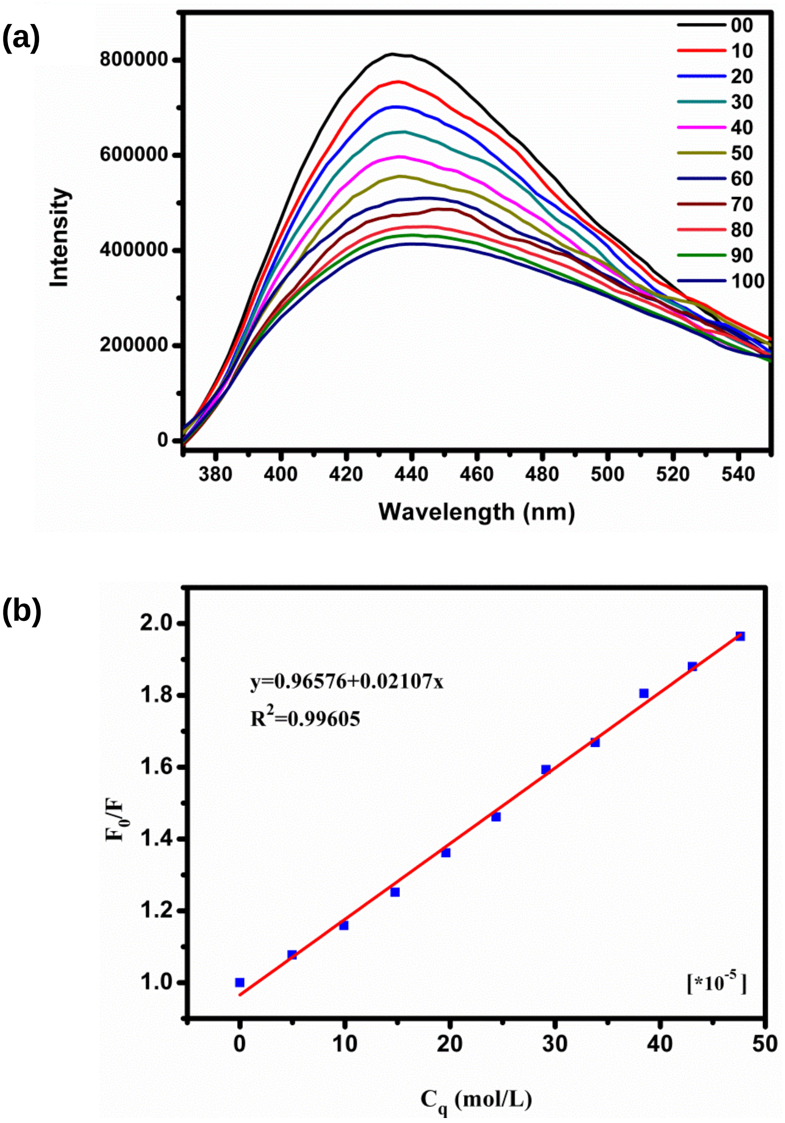

Supplementary Figure 27 │ Fluorescence quenching spectra of InP-1 upon the addition of benzophenone (a) and the corresponding simulated Stern-Volmer curve (b).

Characterization of the *β*–Adduct Product

4-(3-Oxopropyl)benzonitrile

^{1}H NMR (400 MHz, $CDCl_3$) δ 9.82 (s, 1H), 7.59 (d, J = 8.0 Hz, 2H), 7.31 (d, J = 8.0 Hz, 2H), 3.01 (t, J = 7.3 Hz, 2H), 2.83 (t, J = 7.3 Hz, 2H).

^{13}C NMR (126 MHz, $CDCl_3$) δ 200.34, 146.28, 132.61, 129.41, 119.01, 110.58, 44.72, 28.26.

HRMS (EI) exact mass: 159.0682 (Calc. 159.0684)

GC (50 °C 2 min, 15 °C/min, 300 °C) t_R (*β*-adduct product) = 11.15 min, t_R (starting aromatic nitrile) = 10.02 min, t_R (starting aldehyde) = 2.56 min

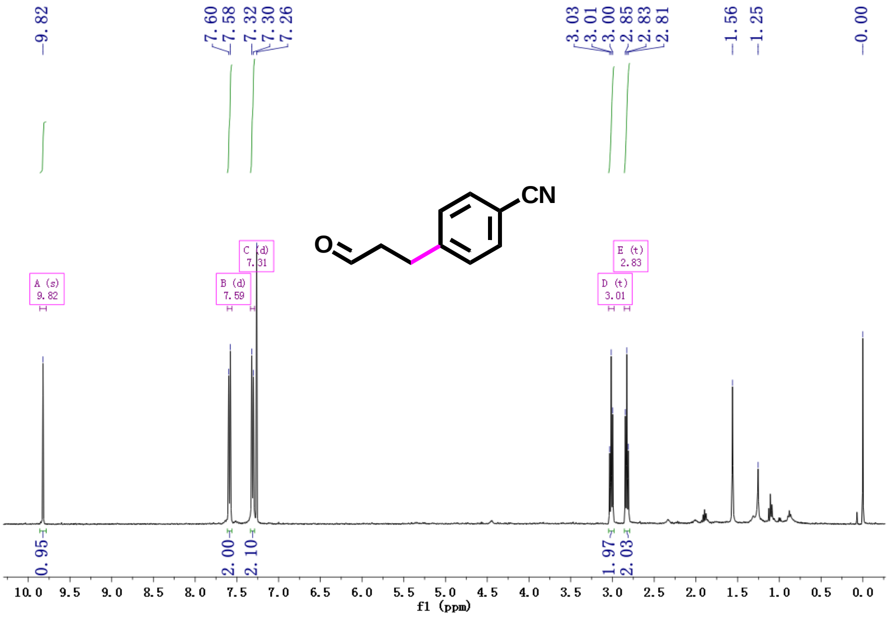

Supplementary Figure 28 │ ^{1}H NMR spectrum of 4-(3-oxopropyl)benzonitrile.

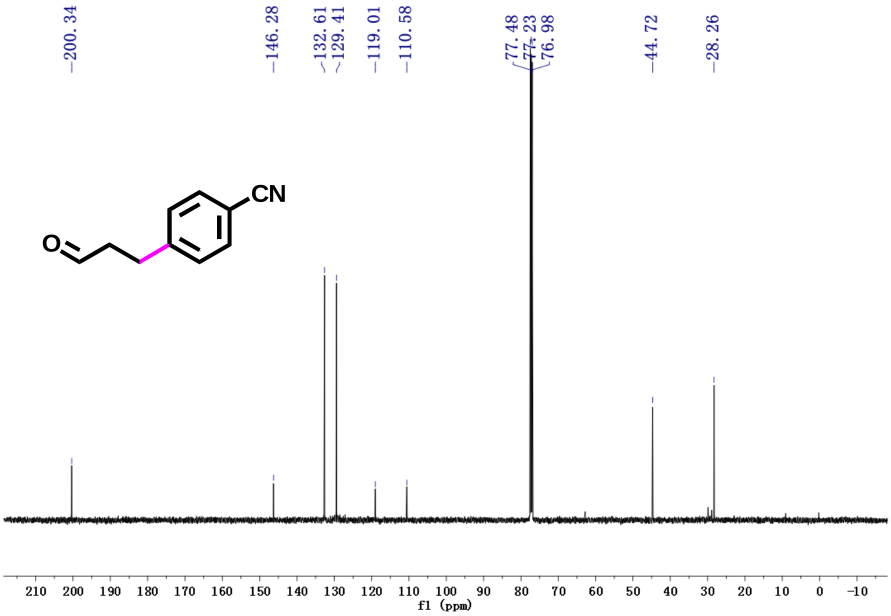

Supplementary Figure 29 │ ^{13}C NMR spectrum of 4-(3-oxopropyl)benzonitrile.

4-(1-Oxopentan-3-yl)benzonitrile

^{1}H NMR (400 MHz, $CDCl_3$) δ 9.68 (s, 1H), 7.60 (d, J = 7.6 Hz, 2H), 7.30 (d, J = 7.8 Hz, 2H), 3.16 (d, J = 7.8 Hz, 1H), 2.77 (t, J = 6.2 Hz, 2H), 1.73 (dd, J = 13.7, 7.0 Hz, 1H), 1.67 – 1.57 (m, 1H), 0.80 (t, J = 7.3 Hz, 3H).

^{13}C NMR (126 MHz, $CDCl_3$) δ 200.65, 149.73, 132.66, 128.64, 119.02, 110.78, 50.05, 41.74, 29.36, 11.98.

HRMS (EI) exact mass: 187.1003 (Calc. 187.0997).

GC (50 °C 2 min, 15 °C/min, 300 °C) t_R (β-adduct product) = 11.27 min, t_R (starting aromatic nitrile) = 10.02 min, t_R (starting aldehyde) = 5.06 min

HPLC Chiral pak AS-H, *i*PrOH/hexane = 15:85, flow rate = 1.0 mL/min, 235 nm, t_R = 17.6 min; t_R = 21.7 min.

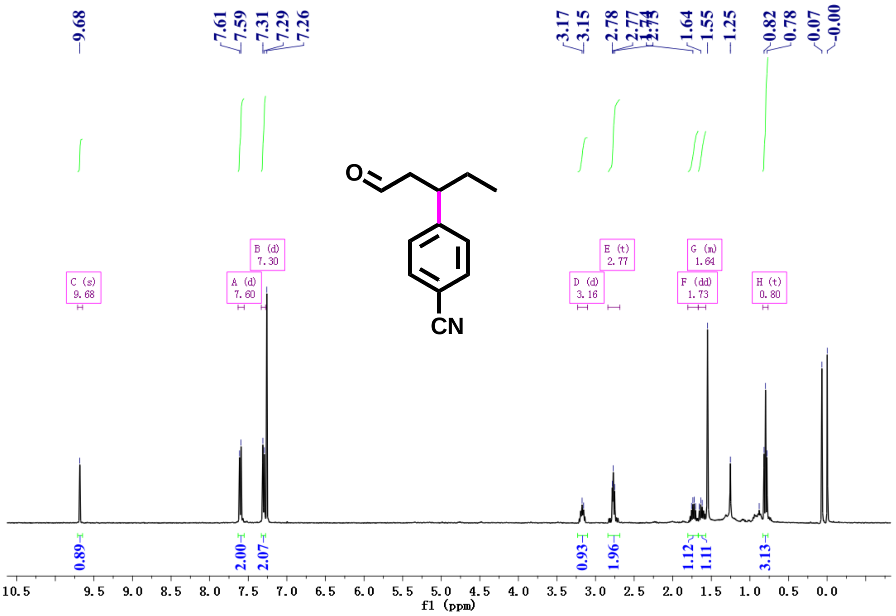

Supplementary Figure 30 | ^{1}H NMR spectrum of 4-(1-oxopentan-3-yl)benzonitrile.

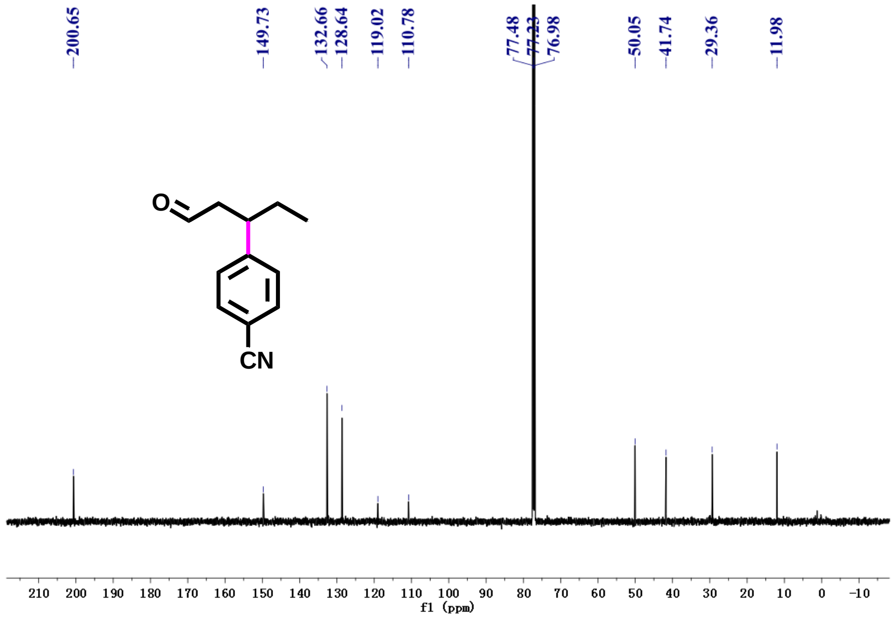

Supplementary Figure 31 | ^{13}C NMR spectrum of 4-(1-oxopentan-3-yl)benzonitrile.

4-(1-Oxooctan-3-yl)benzonitrile

^{1}H NMR (400 MHz, $CDCl_3$) δ 9.67 (s, 1H), 7.60 (d, J = 7.9 Hz, 2H), 7.30 (d, J = 7.9 Hz, 2H), 3.32 – 3.19 (m, 1H), 2.83 – 2.66 (m, 2H), 1.69 – 1.56 (m, 2H), 1.35 – 1.13 (m, 6H), 0.83 (t, J = 6.3 Hz, 3H).

^{13}C NMR (126 MHz, $CDCl_3$) δ 200.63, 150.06, 132.67, 128.58, 119.00, 110.76, 50.43, 40.12, 36.36, 31.76, 27.07, 22.60, 14.13.

HRMS (EI) exact mass: 229.1471 (Calc. 229.1467).

GC (50 °C 2 min, 15 °C/min, 300 °C) t_R (*β*-adduct product) = 12.12 min, t_R (starting aromatic nitrile) = 10.02 min, t_R (starting aldehyde) = 6.19 min

HPLC Chiral pak AS-H, *i*PrOH/hexane = 15:85, flow rate = 1.0 mL/min, 236 nm, t_R = 17.1 min; t_R = 19.6 min.

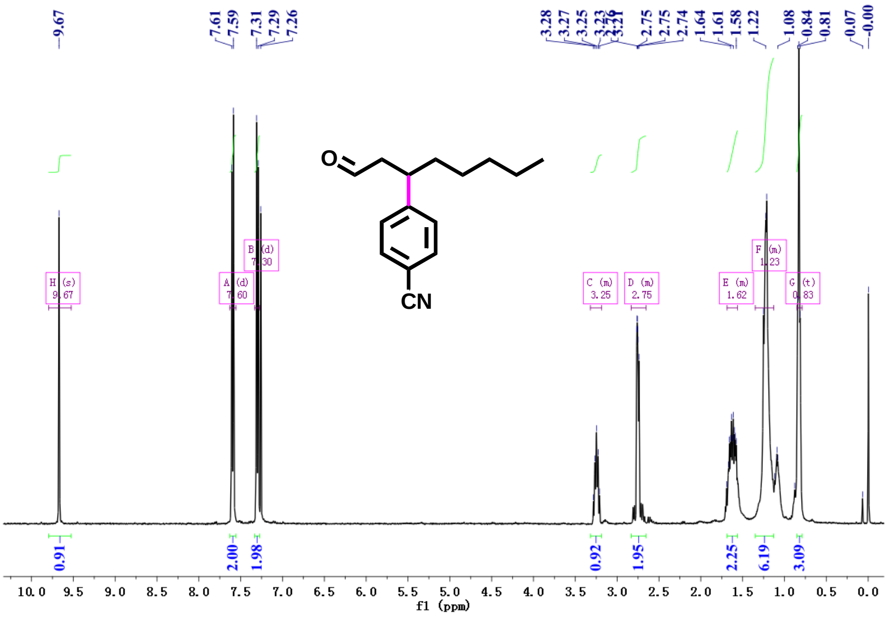

**Supplementary Figure 32 | ** ^{1}H NMR spectrum of 4-(1-oxooctan-3-yl)benzonitrile.

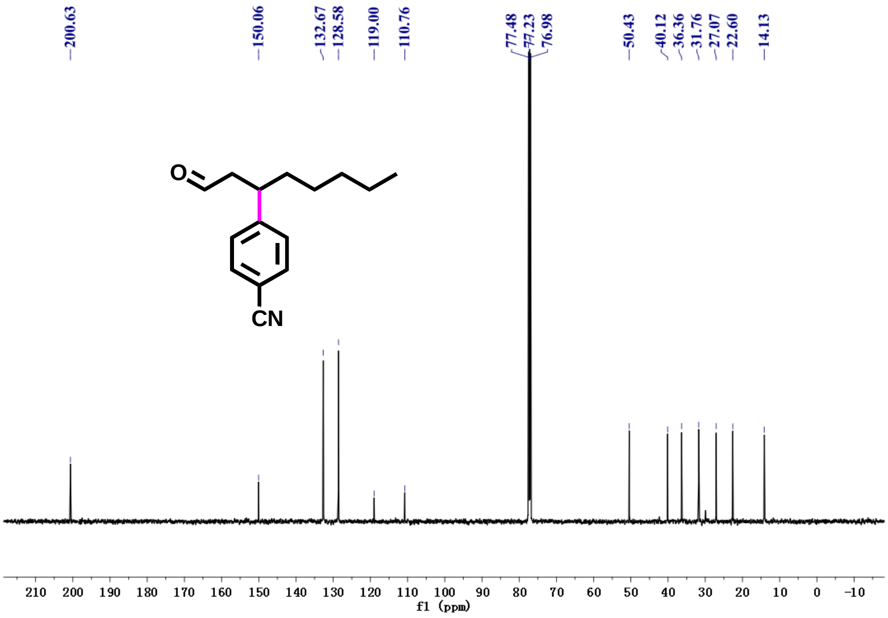

**Supplementary Figure 33 | ** ^{13}C NMR spectrum of 4-(1-oxooctan-3-yl)benzonitrile.

(Z)-4-(1-Oxonon-6-en-3-yl)benzonitrile

^{1}H NMR (400 MHz, $CDCl_3$) δ 9.67 (s, 1H), 7.61 (d, *J* = 8.0 Hz, 2H), 7.31 (d, *J* = 8.0 Hz, 2H), 5.37 (dd, *J* = 17.3, 7.6 Hz, 1H), 5.24 (dd, *J* = 17.7, 7.3 Hz, 1H), 3.39 – 3.16 (m, 1H), 2.76 (d, *J* = 7.4 Hz, 2H), 1.93 – 1.64 (m, 6H), 0.90 (t, *J* = 7.6 Hz, 3H).

^{13}C NMR (126 MHz, $CDCl_3$) δ 200.41, 149.65, 133.08, 132.69, 128.67, 127.61, 118.97, 110.88, 50.43, 39.53, 36.25, 24.87, 20.74, 14.39.

HRMS (EI) exact mass: 241.1481 (Calc. 241.1467).

GC (50 °C 2 min, 15 °C/min, 300 °C) t_R (*β*-adduct product) = 14.63 min, t_R (starting aromatic nitrile) = 10.02 min, t_R (starting aldehyde) = 7.18 min

HPLC Chiral pak AS-H, *i*PrOH/hexane = 20:80, flow rate = 1.0 mL/min, 254 nm, t_R = 11.4 min; t_R = 12.9 min.

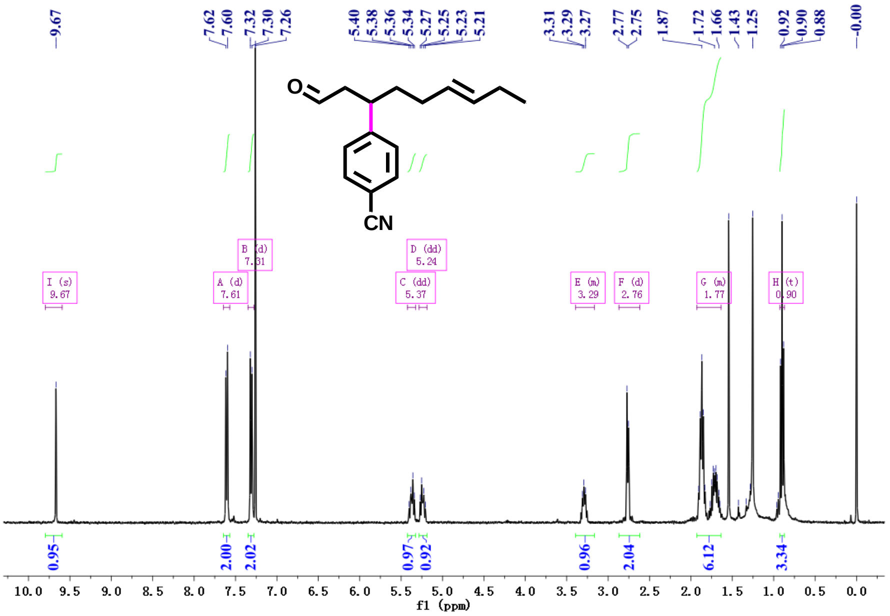

Supplementary Figure 34 │ ^{1}H NMR spectrum of (Z)-4-(1-oxonon-6-en-3-yl)benzonitrile.

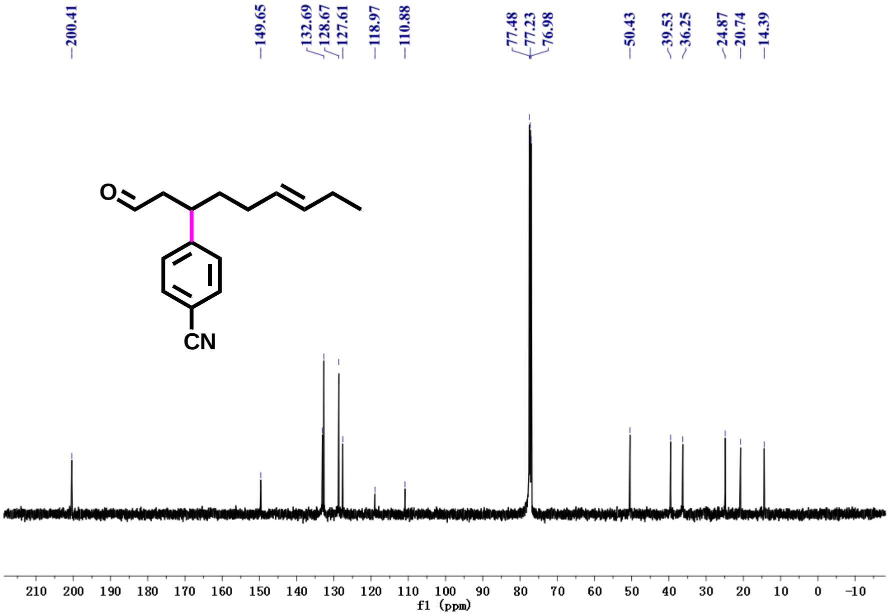

Supplementary Figure 35 │ ^{13}C NMR spectrum of (Z)-4-(1-oxonon-6-en-3-yl)benzonitrile.

4-(3-Hydroxy-1-phenylpropyl)benzonitrile

^{1}H NMR (400 MHz, $CDCl_3$) δ 7.55 (t, *J* = 7.2 Hz, 2H), 7.36 (d, *J* = 8.1 Hz, 2H), 7.33 – 7.27 (m, 2H), 7.22 (d, *J* = 7.9 Hz, 3H), 4.23 (t, *J* = 7.9 Hz, 1H), 3.59 (t, *J* = 6.3 Hz, 2H), 2.40 – 2.20 (m, 2H).

^{13}C NMR (126 MHz, $CDCl_3$) δ 150.46, 143.03, 132.56, 129.04, 128.90, 128.08, 127.09, 119.06, 110.39, 60.64, 47.45, 37.91.

HRMS (EI) exact mass: 237.1165 (Calc. 237.1154).

GC (50 °C 2 min, 15 °C/min, 300 °C) t_R (*β*-adduct product) = 16.42 min, t_R (starting aromatic nitrile) = 10.02 min, t_R (starting aldehyde) = 8.73 min

HPLC Chiral pak AS-H, *i*PrOH/hexane = 15:85, flow rate = 1.1 mL/min, 236 nm, t_R = 18.6 min; t_R = 21.8 min.

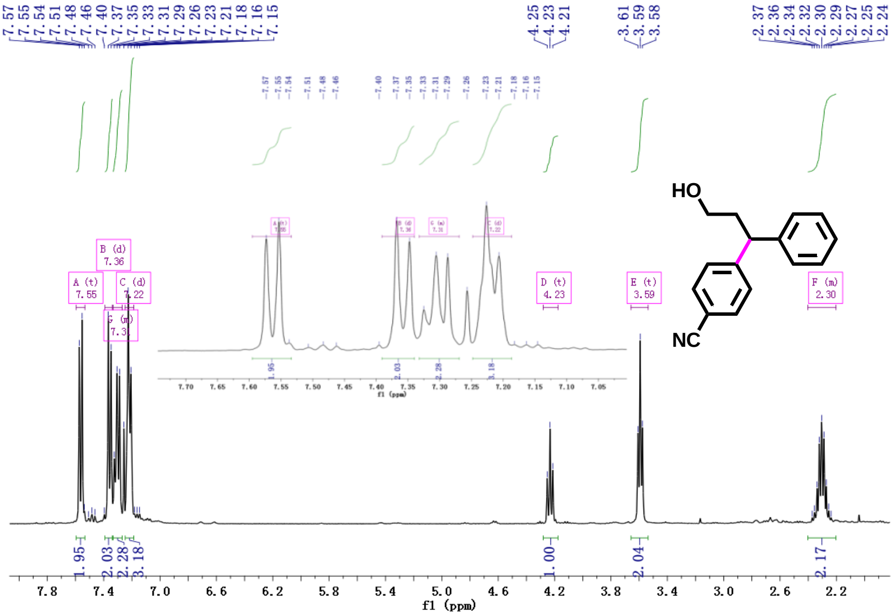

Supplementary Figure 36 | ^{1}H NMR spectrum of 4-(3-hydroxy-1-phenylpropyl)benzonitrile.

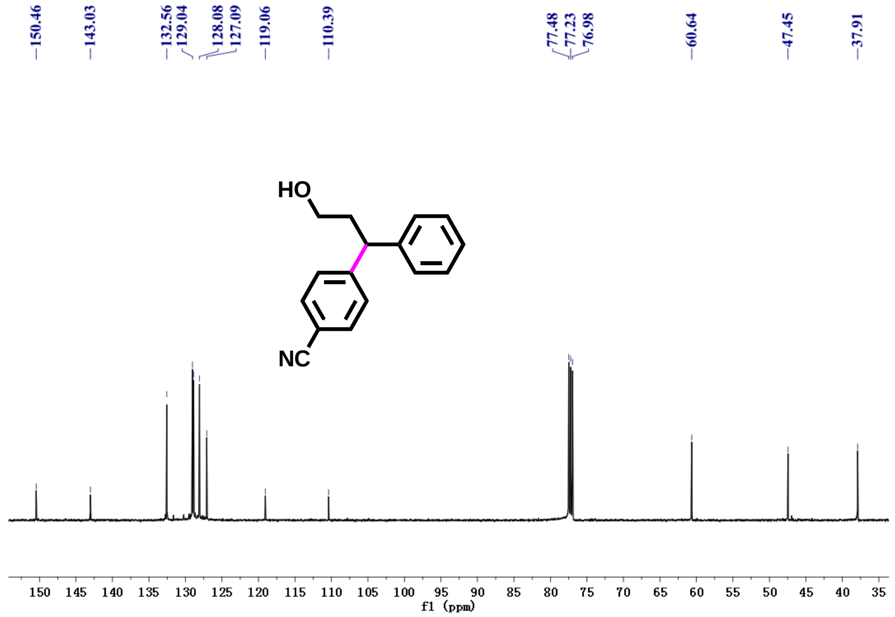

Supplementary Figure 37 | ^{13}C NMR spectrum of 4-(3-hydroxy-1-phenylpropyl)benzonitrile.

4-(3-Hydroxy-1-(4-methoxyphenyl)propyl)benzonitrile

^{1}H NMR (400 MHz, $CDCl_3$) δ 7.56 (d, J = 7.7 Hz, 2H), 7.34 (d, J = 7.9 Hz, 2H), 7.13 (d, J = 8.3 Hz, 2H), 6.84 (d, J = 8.3 Hz, 2H), 4.17 (d, J = 7.8 Hz, 1H), 3.78 (d, J = 3.2 Hz, 3H), 3.59 (t, J = 6.3 Hz, 2H), 2.27 (dd, J = 6.9, 3.2 Hz, 2H).

^{13}C NMR (126 MHz, $CDCl_3$) δ 158.64, 150.88, 135.04, 132.55, 129.03, 128.78, 119.13, 114.42, 110.24, 60.70, 55.46, 46.60, 38.04.

HRMS (EI) exact mass: 267.1262 (Calc. 267.1259).

GC (50 °C 2 min, 15 °C/min, 300 °C) t_R (β-adduct product) = 18.66 min, t_R (starting aromatic nitrile) = 10.02 min, t_R (starting aldehyde) = 11.15 min

HPLC Chiral pak AS-H, *i*PrOH/hexane = 20:80, flow rate = 1.1 mL/min, 235 nm, t_R = 19.4 min; t_R = 23.6 min.

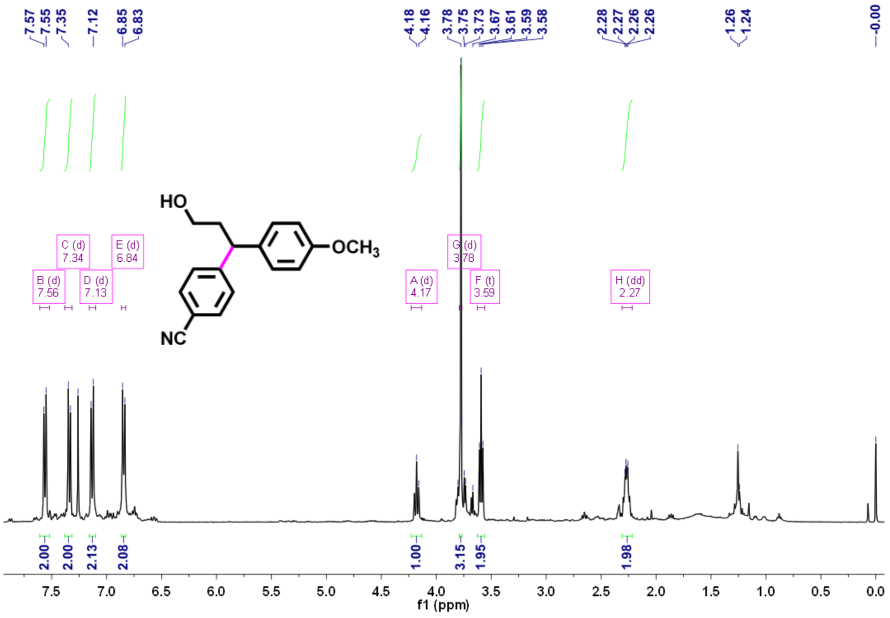

Supplementary Figure 38 | ^{1}H NMR of 4-(3-hydroxy-1-(4-methoxyphenyl)propyl)benzonitrile.

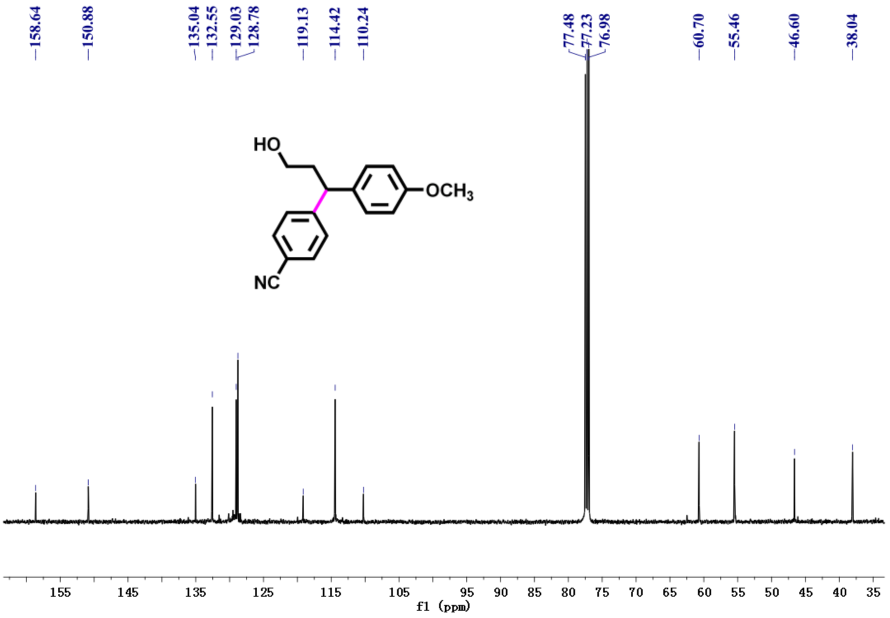

Supplementary Figure 39 | ^{13}C NMR of 4-(3-hydroxy-1-(4-methoxyphenyl)propyl)benzonitrile.

3-(Hydroxydiphenylmethyl)cyclohexan-1-one

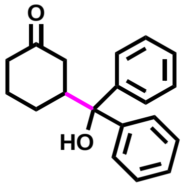

GC-MS (50 °C 3 min, 15 °C/min, 300 °C) t_R (β-adduct product) = 18.02 min and 17.43 min (hemiacetal), t_R (benzophenone) = 12.89 min, t_R (internal standard) = 10.76 min, t_R (DABCO) = 7.00 min, t_R (cyclohexanone) = 5.35 min, t_R (cyclohexanone dimer) = 14.37 min.

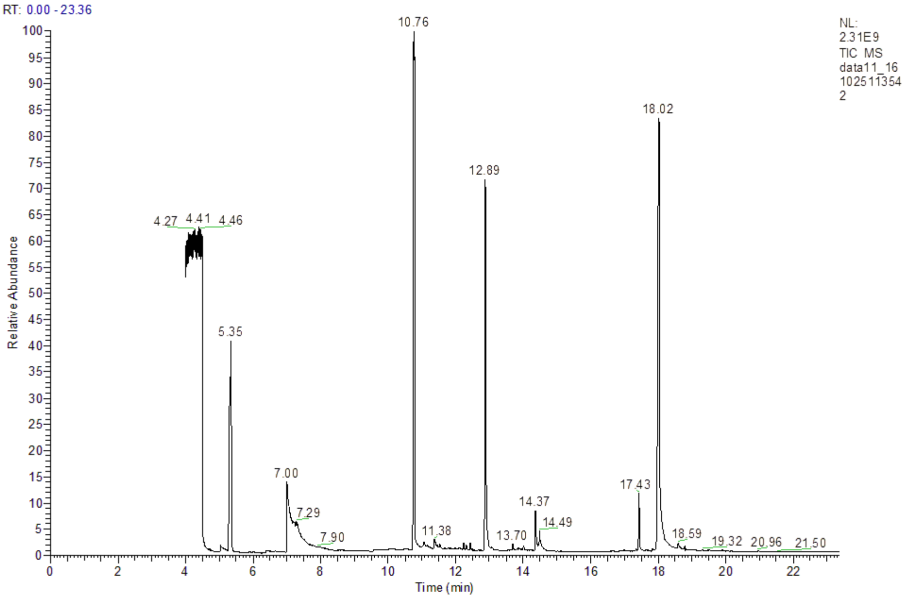

Supplementary Figure 40 │ GC-MS of photocatalytic β-functionalization of cyclohexanone reaction catalyzed by **InP**-1.

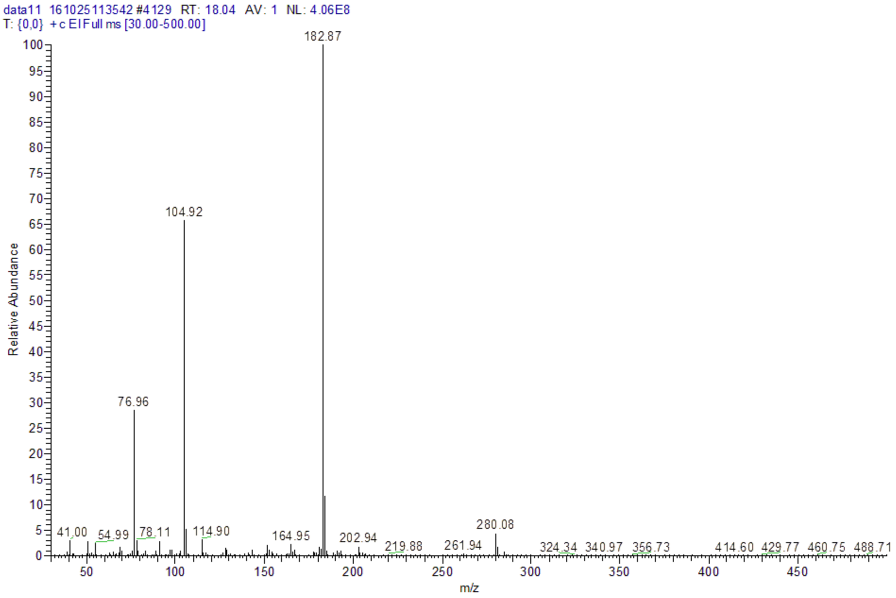

Supplementary Figure 41 │ EI-MS of 3-(hydroxydiphenylmethyl)cyclohexan-1-one.

^{1}H NMR (400 MHz, $CDCl_3$) δ 7.47–7.45 (dd, 2H), 7.42–7.39 (dd, 2H), 7.34 – 7.29 (m, 4H), 7.25 – 7.17 (m, 2H), 2.92 (tdd, 1H, *J* = 11.3, 5.4, 3.1 Hz), 2.37 (dd, 1H, *J* = 14.8, 13.1 Hz), 2.32 – 2.21 (m, 3H), 2.08 (dtd, *J* = 9.6, 6.3, 3.1 Hz, 1H), 1.88 – 1.76 (m, 1H), 1.69 (ddd, 1H, *J* = 13.2, 10.8, 4.5 Hz), 1.52 – 1.44 (m, 1H)[11].

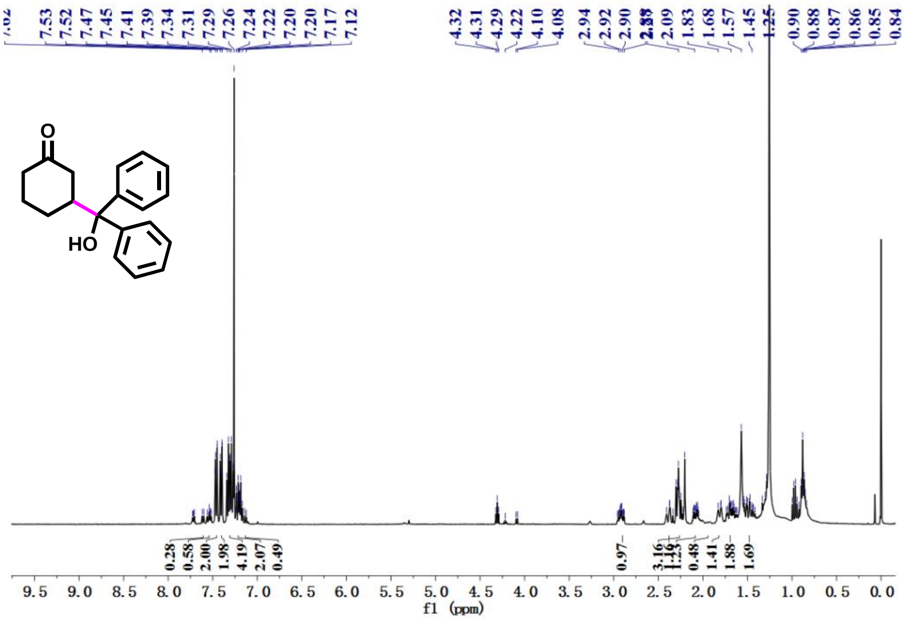

Supplementary Figure 42 │ ^{1}H NMR spectrum of 3-(hydroxydiphenylmethyl)cyclohexan-1-one.

3-(Hydroxydiphenylmethyl)-4-methylcyclohexan-1-one

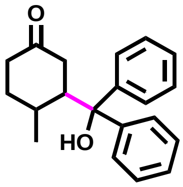

GC-MS (50 °C 3 min, 15 °C/min, 300 °C) t_R (*β*-adduct product) = 17.77 min and 18.07 min (hemiacetal), t_R (benzophenone) = 12.88 min, t_R (internal standard) = 10.76 min, t_R (DABCO) = 7.01 min, t_R (4-methylcyclohexanone) = 6.22 min.

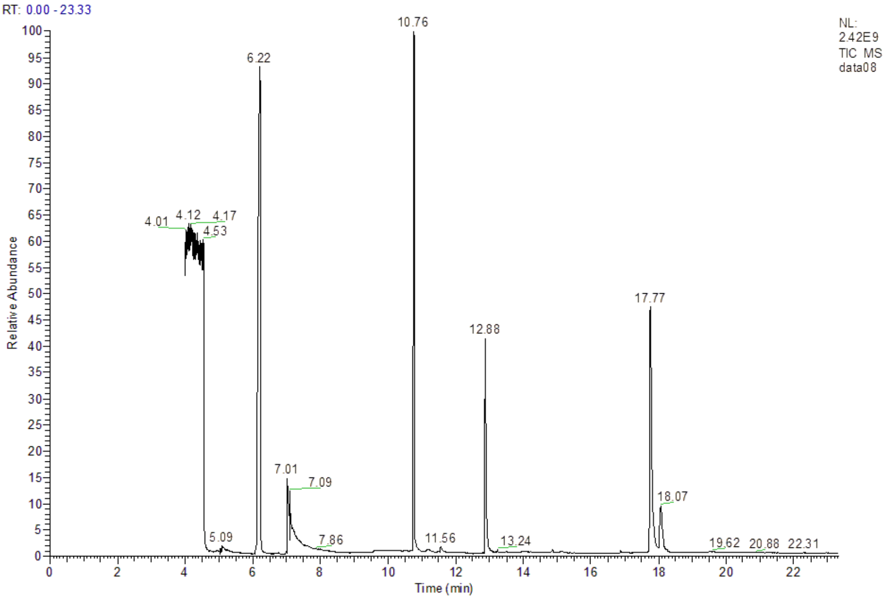

Supplementary Figure 43 │ GC-MS of photocatalytic *β*-functionalization of 4-methylcyclohexanone reaction catalyzed by **InP**-1.

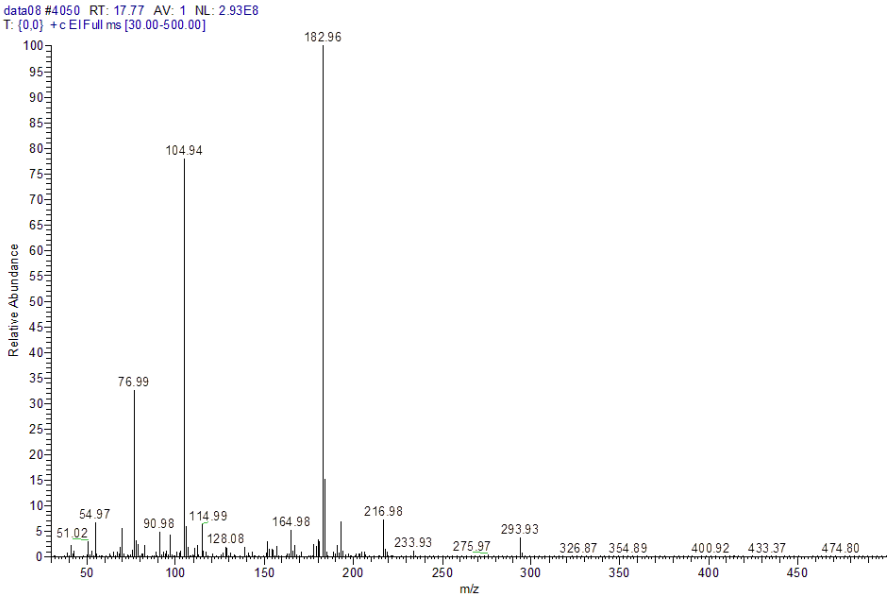

Supplementary Figure 44 │ EI-MS of 3-(hydroxydiphenylmethyl)-4-methylcyclohexan-1-one.

^{1}H NMR (400 MHz, $CDCl_3$) δ 7.71 – 7.45 (m, 4H), 7.36 – 7.28 (m, 4H), 7.22 – 7.14 (m, 2H), 3.07 (s, 1H), 2.52 (dd, *J* = 45.4, 36.9 Hz, 1H), 2.31 (d, *J* = 14.4 Hz, 1H), 2.05 – 1.73 (m, 4H), 1.39 – 1.13 (m, 2H), 1.13 – 0.96 (m, 3H)[11].

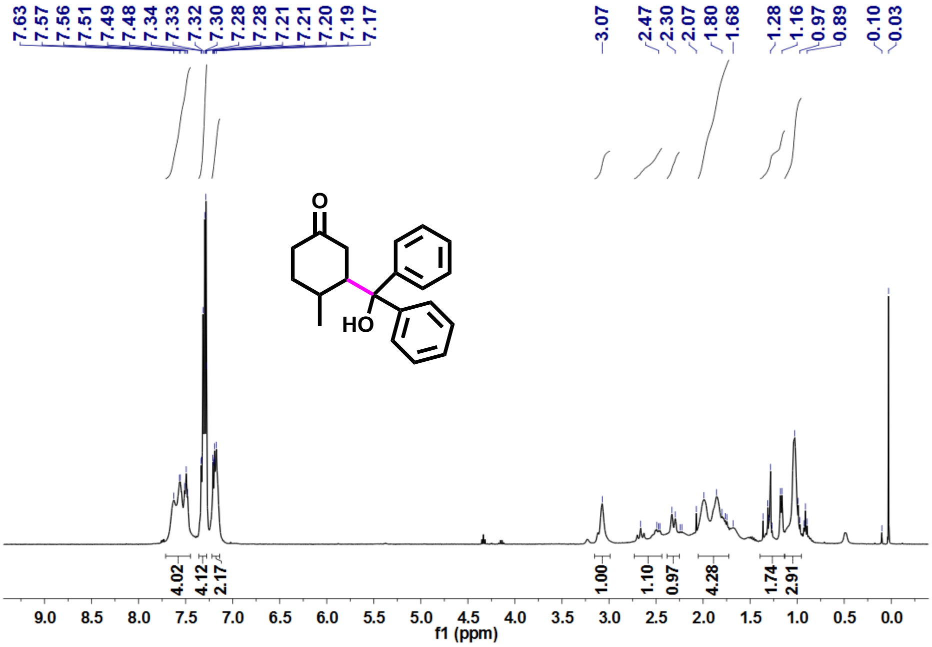

Supplementary Figure 45 │ ^{1}H NMR of 3-(hydroxydiphenylmethyl)-4-methylcyclohexan-1-one.

3-(Hydroxydiphenylmethyl)-5-methylcyclohexan-1-one

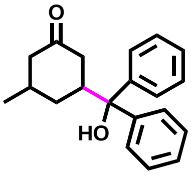

GC-MS (50 °C 3 min, 15 °C/min, 300 °C) t_R (β-adduct product) = 18.13 min and 17.94 min (hemiacetal), t_R (benzophenone) = 12.90 min, t_R (internal standard) = 10.77 min, t_R (DABCO) = 6.97 min, t_R (3-methylcyclohexanone) = 6.11 min.

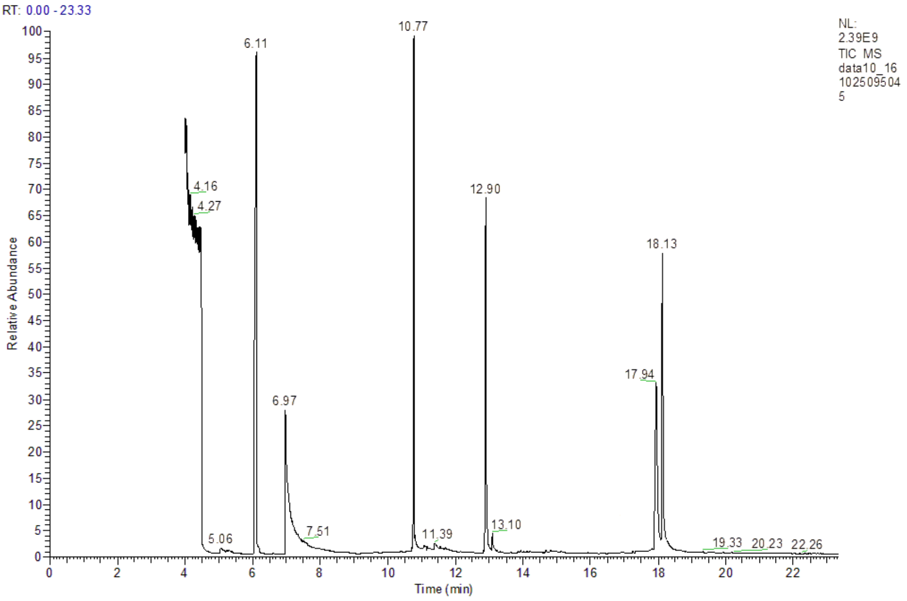

Supplementary Figure 46 │ GC-MS of photocatalytic β-functionalization of 3-methylcyclohexanone reaction catalyzed by **InP**-1.

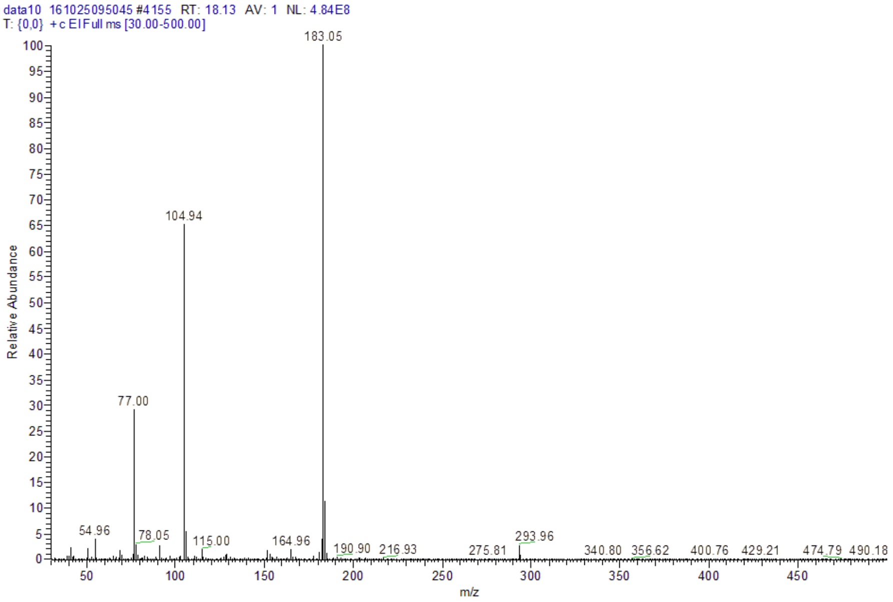

Supplementary Figure 47 │ EI-MS of 3-(hydroxydiphenylmethyl)-5-methylcyclohexan-1-one.

^{1}H NMR (400 MHz, $CDCl_3$) δ 7.49 – 7.44 (m, 2H), 7.42 – 7.38 (m, 2H), 7.33 (t, *J* = 7.7 Hz, 2H), 7.30 (d, *J* = 7.4 Hz, 2H), 7.23 (t, *J* = 7.3 Hz, 1H), 7.18 (t, *J* = 7.3 Hz, 1H), 2.99 – 2.83 (m, 1H), 2.38 (d, *J* = 10.3 Hz, 1H), 2.26 – 2.19 (m, 2H), 1.92 (ddd, *J* = 16.1, 9.6, 8.2 Hz, 2H), 1.78 (d, *J* = 13.2 Hz, 1H), 1.29 – 1.23 (m, 2H), 0.98 (t, *J* = 7.5 Hz, 3H)[11].

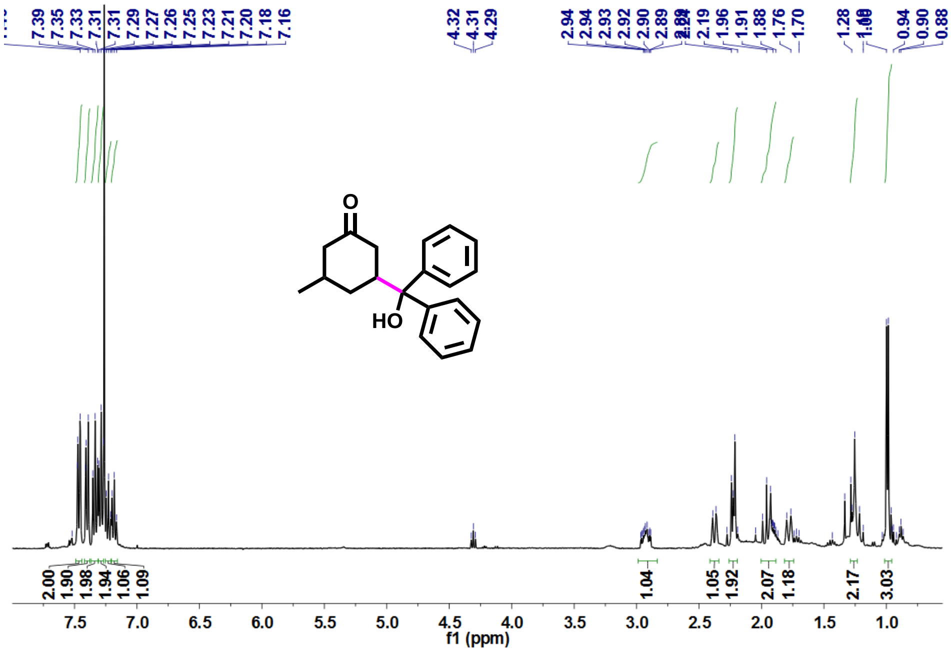

Supplementary Figure 48 │ ^{1}H NMR of 3-(hydroxydiphenylmethyl)-5-methylcyclohexan-1-one.

5-(Hydroxydiphenylmethyl)-3,3-dimethylcyclohexan-1-one

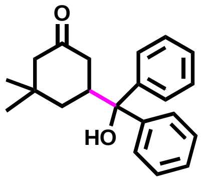

GC-MS (50 °C 3 min, 15 °C/min, 300 °C) t_R (β-adduct product) = 18.12 min, t_R (benzophenone) = 12.89 min, t_R (internal standard) = 10.76 min, t_R (DABCO) = 7.04 min, t_R (3,3-dimethylcyclohexanone) = 6.70 min, t_R (3,3-dimethylcyclohexanone dimer) = 15.18 min.

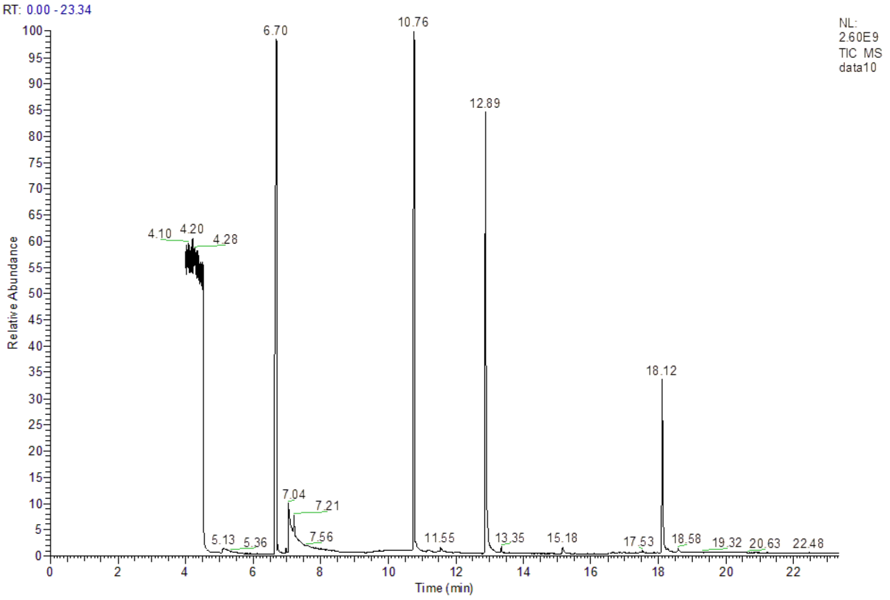

Supplementary Figure 49 │ GC-MS of photocatalytic β-functionalization of 3,3-dimethylcyclohexanone reaction catalyzed by **InP**-1.

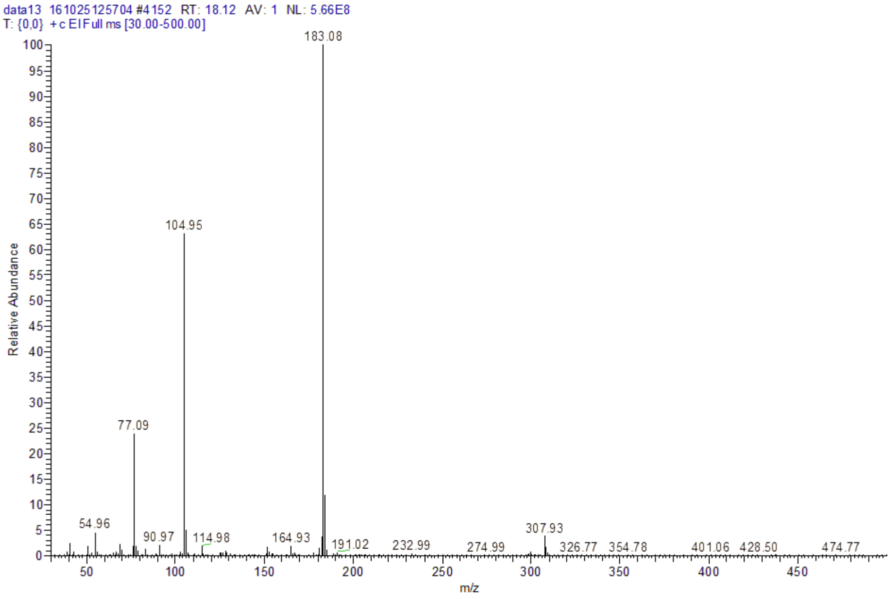

Supplementary Figure 50 │ EI-MS of 5-(hydroxydiphenylmethyl)-3,3-dimethylcyclohexan-1-one.

^{1}H NMR (400 MHz, $CDCl_3$) δ 7.47 (dd, J = 8.4, 1.1 Hz, 2H), 7.40 (dd, J = 8.4, 1.1 Hz, 2H), 7.34 (dd, J = 10.5, 5.0 Hz, 2H), 7.31 – 7.26 (m, 2H), 7.25 – 7.20 (m, 1H), 7.18 (dd, J = 10.4, 4.2 Hz, 1H), 3.19 – 2.99 (m, 1H), 2.25 (d, J = 13.5 Hz, 1H), 2.20 (s, 1H), 2.17 (s, 1H), 2.13 (dt, J = 12.2, 4.3 Hz, 2H), 1.52 (d, J = 11.2 Hz, 1H), 1.03 (s, 3H), 0.99 (d, J = 5.2 Hz, 3H)[11].

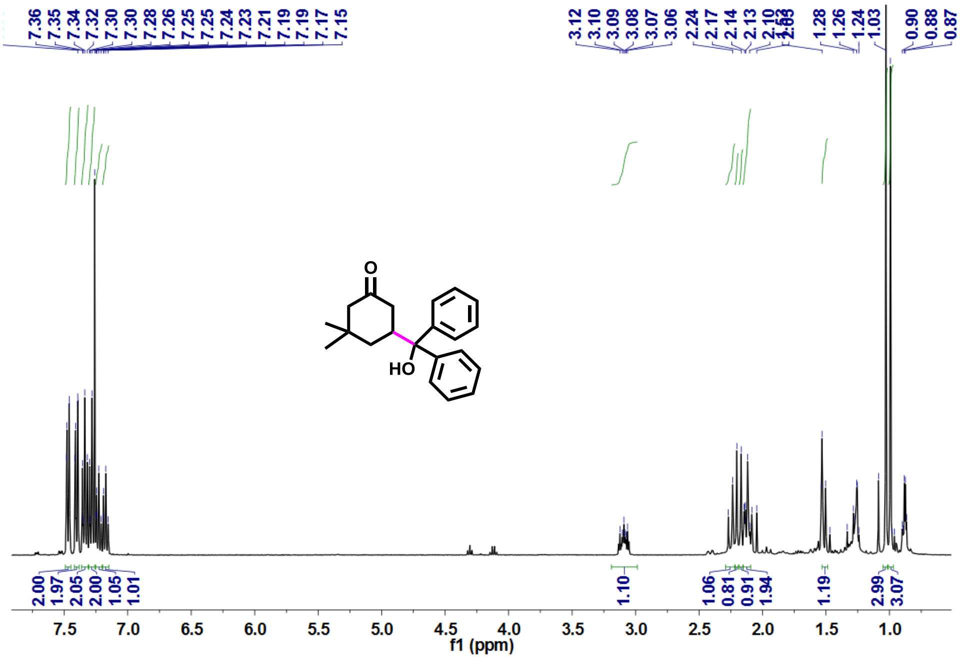

Supplementary Figure 51 | ^{1}H NMR of 5-(hydroxydiphenylmethyl)-3,3-dimethylcyclohexan-1-one.

3-(Hydroxydiphenylmethyl)cyclopentan-1-one

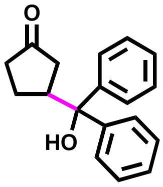

GC-MS (50 °C 3 min, 15 °C/min, 300 °C) t_R (β-adduct product) = 17.60 min, t_R (benzophenone) = 12.88 min, t_R (internal standard) = 10.75 min, t_R (DABCO) = 7.01 min, t_R (cyclopentanone dimer) = 12.06 min.

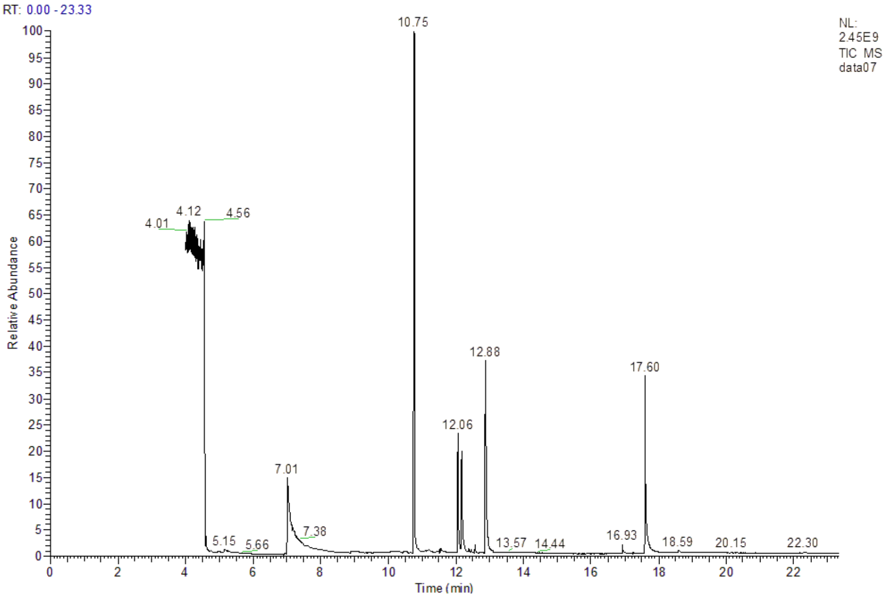

Supplementary Figure 52 │ GC-MS of photocatalytic β-functionalization of cyclopentanone reaction catalyzed by **InP**-1.

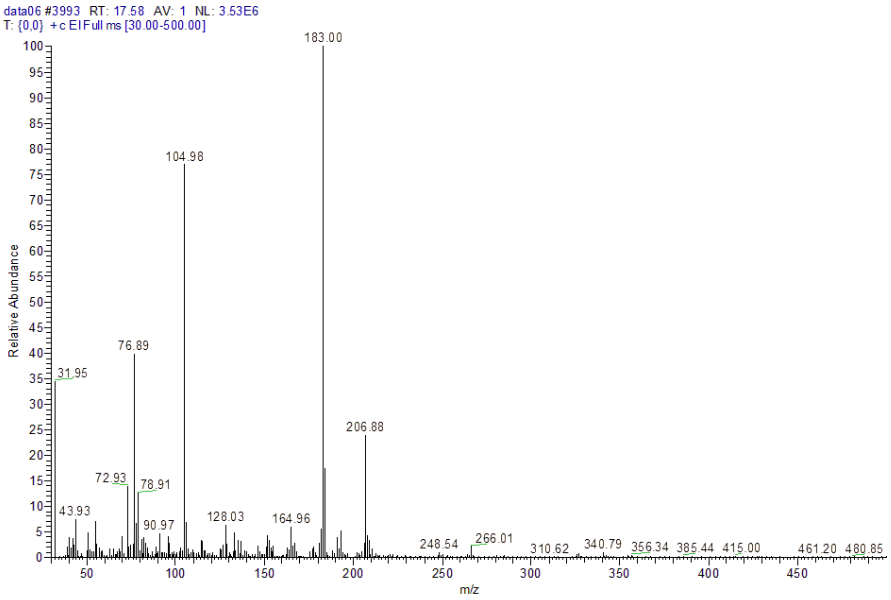

Supplementary Figure 53 │ EI-MS of 3-(hydroxydiphenylmethyl)cyclopentan-1-one.

^{1}H NMR (400 MHz, $CDCl_3$) δ 7.49 – 7.44 (m, 2H), 7.44 – 7.40 (m, 1H), 7.34 (t, *J* = 5.1 Hz, 1H), 7.30 (t, *J* = 5.1 Hz, 1H), 7.22 (dd, *J* = 15.0, 7.5 Hz, 1H), 3.43 – 3.33 (m, 1H), 2.33 (dd, *J* = 7.7, 5.1 Hz, 1H), 2.22 (dt, *J* = 18.7, 5.9 Hz, 2H), 1.92 – 1.83 (m, 1H)[11].

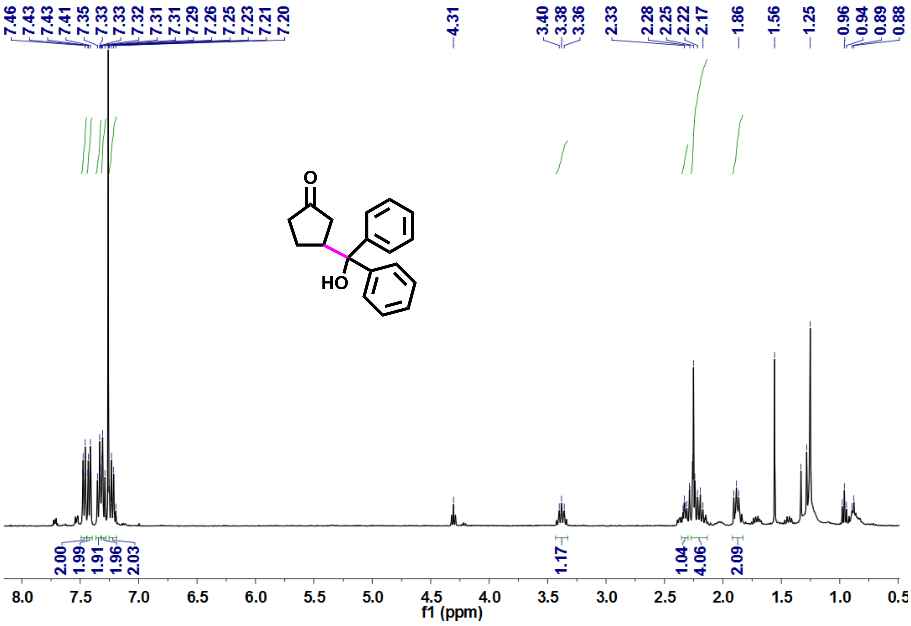

Supplementary Figure 54 │ ^{1}H NMR of 3-(hydroxydiphenylmethyl)cyclopentan-1-one.

HPLC Chromatograms for the Catalytic Product

(a)

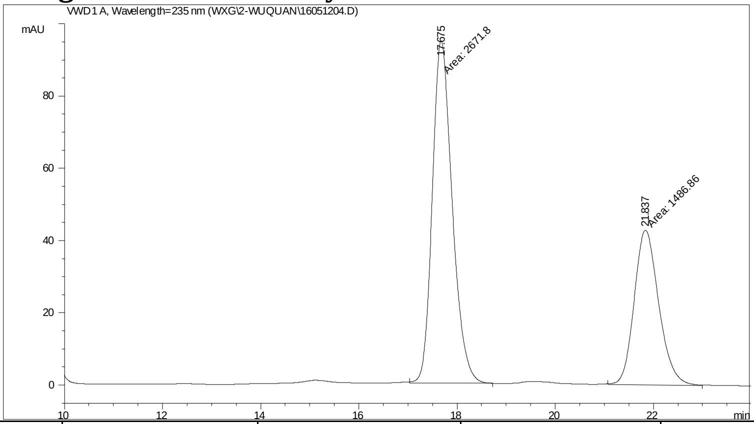

Peak #	RetTime [min]	Area [mAU *s]	Height [mAU]	Area [%]
1	17.675	2671.796	94.804	64.247
2	21.837	1486.858	42.781	35.753
Total	—	4158.654	137.586	100.000

(b)

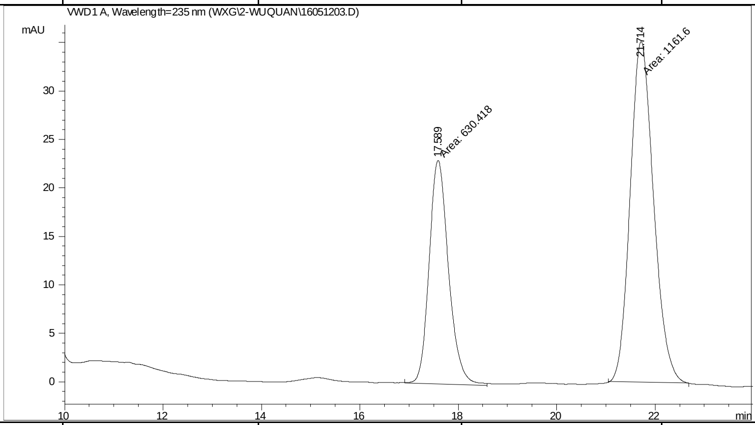

Peak #	RetTime [min]	Area [mAU *s]	Height [mAU]	Area [%]
1	17.589	630.418	23.050	35.179
2	21.714	1161.596	35.135	64.821
Total	—	1792.014	58.184	100.000

(c)

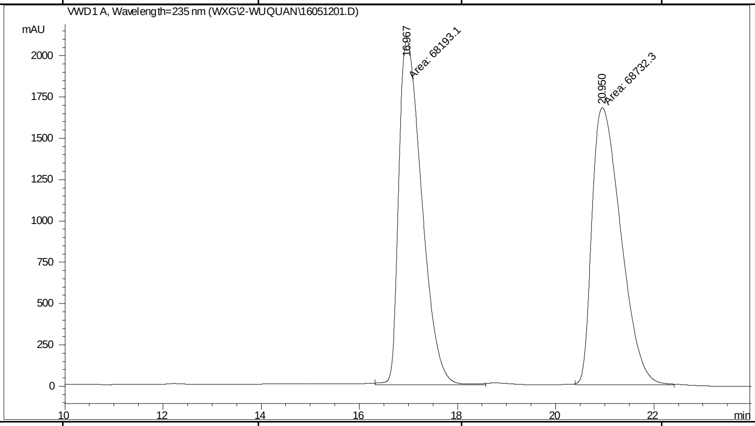

Peak #	RetTime [min]	Area [mAU *s]	Height [mAU]	Area [%]
1	16.967	68193.090	2080.104	49.803
2	20.950	68732.300	1676.214	50.197
Total	—	136925.390	3756.318	100.000

Supplementary Figure 55 │ HPLC chromatograms of 4-(1-oxopentan-3-yl)benzonitrile formed with the catalysis of **InP**-1 (a) and **InP**-2 (b), and HPLC trace of the racemic adduct (c).

(a)

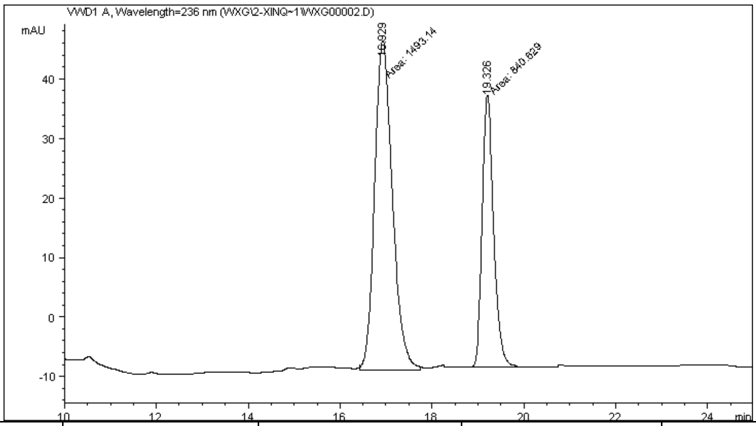

Peak #	RetTime [min]	Area [mAU *s]	Height [mAU]	Area [%]
1	16.929	1493.141	46.116	63.980
2	19.326	840.629	36.915	36.020
Total	—	2333.770	83.031	100.000

(b)

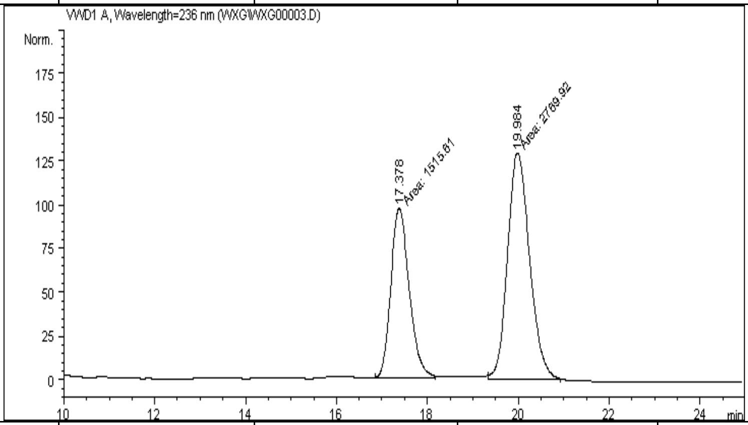

Peak #	RetTime [min]	Area [mAU *s]	Height [mAU]	Area [%]
1	17.378	1515.611	96.991	35.366
2	19.984	2769.921	129.505	64.634
Total	—	4285.532	226.496	100.000

(c)

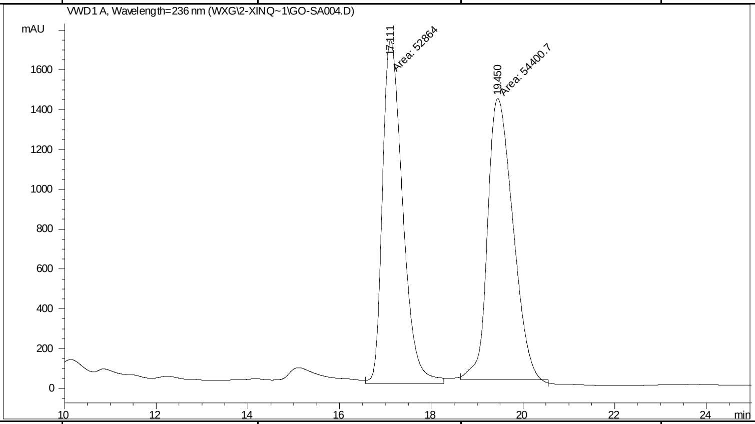

Peak #	RetTime [min]	Area [mAU *s]	Height [mAU]	Area [%]
1	17.111	52863.957	1724.473	49.284
2	19.450	54400.710	1412.948	50.716
Total	—	107264.667	3137.421	100.000

Supplementary Figure 56 │ HPLC chromatogram of 4-(1-oxooctan-3-yl)benzonitrile formed with the catalysis of **InP**-1 (a) and **InP**-2 (b), and HPLC trace of the racemic adduct (c).

(a)

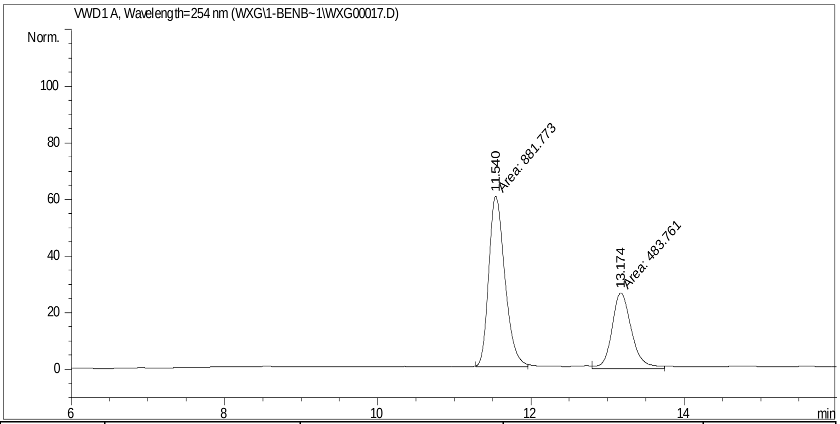

Peak #	RetTime [min]	Area [mAU *s]	Height [mAU]	Area [%]
1	11.540	881.773	60.177	64.5735
2	13.174	483.761	26.866	35.4265
Total	—	1365.534	87.043	100.000

(b)

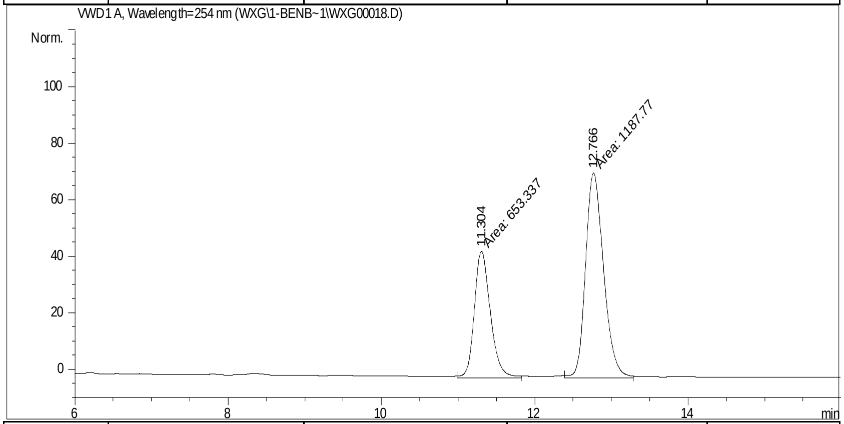

Peak #	RetTime [min]	Area [mAU *s]	Height [mAU]	Area [%]
1	11.304	653.337	42.759	35.4861
2	12.766	1187.772	69.470	64.5139
Total	—	1841.109	112.229	100.000

(c)

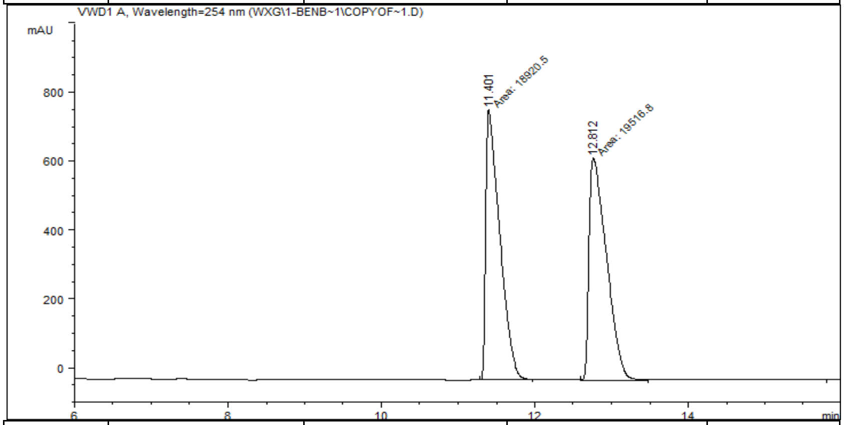

Peak #	RetTime [min]	Area [mAU *s]	Height [mAU]	Area [%]
1	11.401	18920.512	782.336	49.224
2	12.812	19516.837	642.031	50.776
Total	—	38437.349	1424.367	100.000

Supplementary Figure 57 │ HPLC chromatogram of (Z)-4-(1-oxonon-6-en-3-yl)benzonitrile formed with the catalysis of **InP**-1 (a) and **InP**-2 (b), and HPLC trace of the racemic adduct (c).

(a)

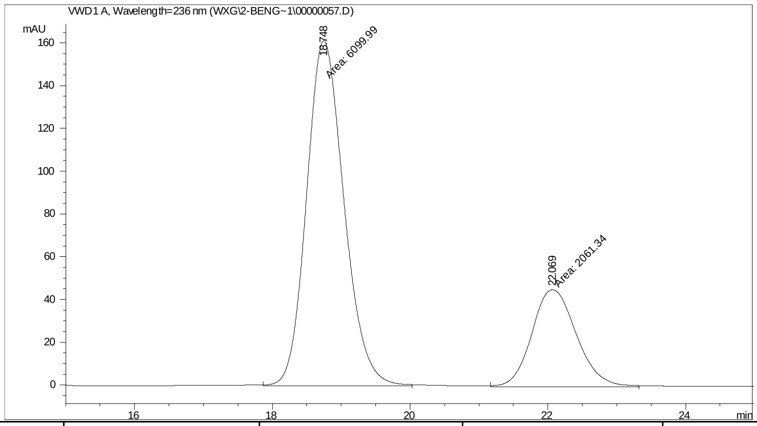

Peak #	RetTime [min]	Area [mAU *s]	Height [mAU]	Area [%]
1	18.748	6099.990	161.496	74.743
2	22.069	2061.339	45.504	25.257
Total	—	8161.329	207.000	100.000

(b)

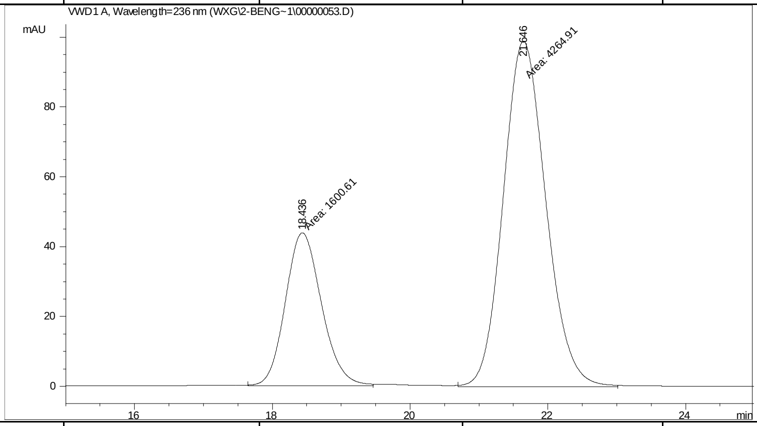

Peak #	RetTime [min]	Area [mAU *s]	Height [mAU]	Area [%]
1	18.436	1600.614	43.832	27.288
2	21.646	4264.915	99.113	72.712
Total	—	5865.529	142.945	100.000

(c)

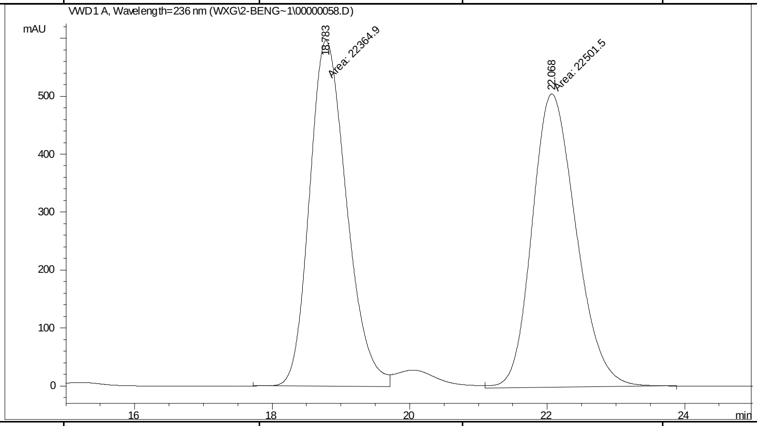

Peak #	RetTime [min]	Area [mAU *s]	Height [mAU]	Area [%]
1	18.783	22364.854	596.904	49.848
2	22.068	22501.470	506.118	50.152
Total	—	44866.324	1103.022	100.000

Supplementary Figure 58 │ HPLC chromatogram of 4-(3-hydroxy-1-phenylpropyl)benzonitrile formed with the catalysis of **InP**-1 (a) and **InP**-2 (b), and HPLC trace of the racemic adduct (c).

(a)

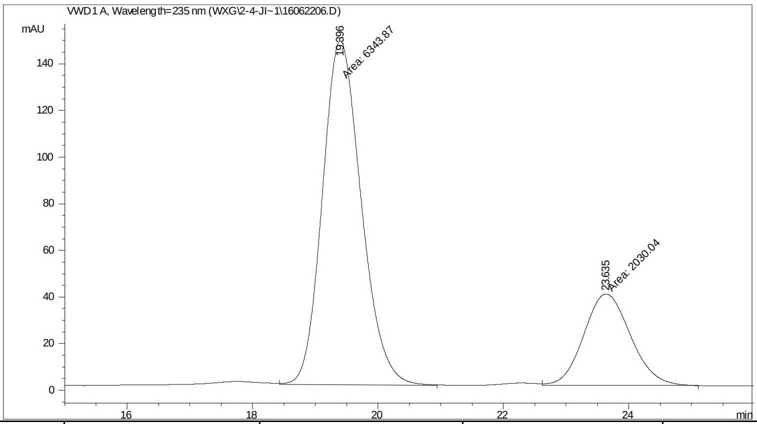

Peak #	RetTime [min]	Area [mAU *s]	Height [mAU]	Area [%]
1	19.396	6343.874	147.532	75.758
2	23.635	2030.041	39.195	24.242
Total	—	8373.915	186.727	100.000

(b)

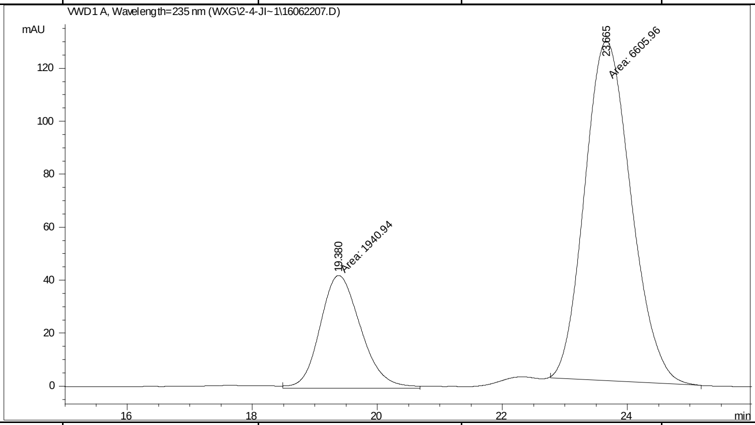

Peak #	RetTime [min]	Area [mAU *s]	Height [mAU]	Area [%]
1	19.380	1940.940	42.621	22.709
2	23.665	6605.962	128.208	77.291
Total	—	8546.902	170.829	100.000

(c)

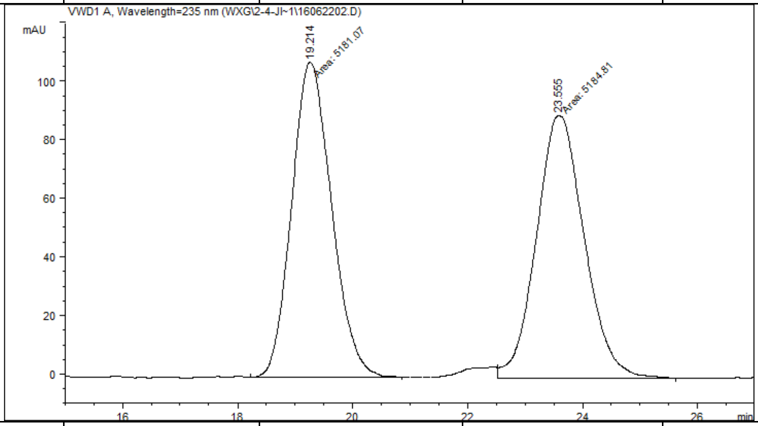

Peak #	RetTime [min]	Area [mAU *s]	Height [mAU]	Area [%]
1	19.214	5181.066	107.514	49.982
2	23.555	5184.809	89.610	50.018
Total	—	10365.875	197.124	100.000

Supplementary Figure 59 │ HPLC chromatogram of 4-(3-hydroxy-1-(4-methoxyphenyl)propyl) benzonitrile formed with the catalysis of **InP**-1 (a) and **InP**-2 (b), and HPLC trace of the racemic adduct (c).

Supplementary References

1. Wang, J., He, C., Wu, P., Wang, J. & Duan, C. An amide-containing metal–organic tetrahedron responding to a spin-trapping reaction in a fluorescent enhancement manner for biological imaging of NO in living cells. *J. Am. Chem. Soc.* **133,** 12402–12405 (2011).

2. Dang, D., Wu, P., He, C., Xie, Z. & Duan, C. Homochiral metal-organic frameworks for heterogeneous asymmetric catalysis. *J. Am. Chem. Soc.* **132,** 14321–14323 (2010).

3. Chae, H. K., Kim, J., Friedrichs, O. D., O'Keeffe, M. & Yaghi, O. M. Design of frameworks with mixed triangular and octahedral building blocks exemplified by the structure of [$Zn_4O(TCA)_2$] having the pyrite topology. *Angew. Chem. Int. Ed.* **42,** 3907–3909 (2003).

4. Wu, P. *et al*. Photoactive chiral metal−organic frameworks for light-driven asymmetric *α*-alkylation of aldehydes. *J. Am. Chem. Soc.* **134,** 14991−14999 (2012).

5. Sheldrick, G. M. SAINT 5.1 ed. (Siemens Industrial Automation Inc., Madison, WI, 1995).

6. SADABS, Empirical Absorption Correction Program (University of Göttingen, Göttingen, Germany, 1997).

7. Sheldrick, G. M. SHELXTL Reference Manual: Version 5.1 (Bruker AXS, Madison, WI, 1997).

8. Sheldrick, G. M. SHELXL-97: Program for Crystal Structure Refinement (University of Göttingen, Göttingen, Germany, 1997).

9. Pirnot, M. T., Rankic, D. A., Martin, D. B. C. & MacMillan, D. W. C. Photoredox activation for the direct *β*-arylation of ketones and aldehydes. *Science* **339,** 1593–1596 (2013).

10. Banerjee, M. *et al*. Postsynthetic modification switches an achiral framework to catalytically active homochiral metal-organic porous materials. *J. Am. Chem. Soc.* **131,** 7524–7525 (2009).

11. Petronijević, F. R., Nappi, M. & MacMillan, D. W. C. Direct *β*-functionalization of cyclic ketones with aryl ketones via the merger of photoredox and organocatalysis. *J. Am. Chem. Soc.* **135,** 18323–18326 (2013).
